# Attomolar DNA detection with chiral nanorod assemblies

**DOI:** 10.1038/ncomms3689

**Published:** 2013-10-28

**Authors:** Wei Ma, Hua Kuang, Liguang Xu, Li Ding, Chuanlai Xu, Libing Wang, Nicholas A. Kotov

**Affiliations:** 1 State Key Lab of Food Science and Technology, School of Food Science and Technology, Jiangnan University, Wuxi, Jiangsu 214122, China; 2 State Key Lab of Food Safety Test (Hunan), Changsha, Hunan 410004, China; 3 Department of Chemical Engineering, University of Michigan, Ann Arbor, Michigan 48109, USA; 4 Department of Materials Science, University of Michigan, Ann Arbor, Michigan 48109, USA; 5 Department of Biomedical Engineering, University of Michigan, Ann Arbor, Michigan 48109, USA; 6 Biointerface Institute, University of Michigan, Ann Arbor, Michigan 48109, USA; 7 These authors contributed equally to this work

## Abstract

Nanoscale plasmonic assemblies display exceptionally strong chiral optical activity. So far, their structural design was primarily driven by challenges related to metamaterials whose practical applications are remote. Here we demonstrate that gold nanorods assembled by the polymerase chain reaction into DNA-bridged chiral systems have promising analytical applications. The chiroplasmonic activity of side-by-side assembled patterns is attributed to a 7–9 degree twist between the nanorod axes. This results in a strong polarization rotation that matches theoretical expectations. The amplitude of the bisignate ‘wave’ in the circular dichroism spectra of side-by-side assemblies demonstrates excellent linearity with the amount of target DNA. The limit of detection for DNA using side-by-side assemblies is as low as 3.7 aM. This chiroplasmonic method may be particularly useful for biological analytes larger than 2–5 nm which are difficult to detect by methods based on plasmon coupling and ‘hot spots’. Circular polarization increases for inter-nanorod gaps between 2 and 20 nm when plasmonic coupling rapidly decreases. Reaching the attomolar limit of detection for simple and reliable bioanalysis of oligonucleotides may have a crucial role in DNA biomarker detection for early diagnostics of different diseases, forensics and environmental monitoring.

## Abstract (web summary)

Nanoscale plasmonic assemblies are known to display exceptionally strong chiral optical activity. Here, the authors assemble gold nanorods into DNA-bridged chiral systems, and demonstrate their high efficiency for DNA detection at very low concentrations.

## Graphical abstract

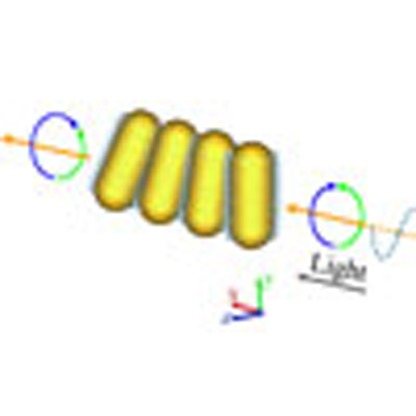

Chirality of nanoparticles (NPs) and their assemblies have recently attracted substantial interest among materials scientists^1^,^2^,^3^,^4^,^5^. There now exist examples of NP assemblies with pyramidal^3^,^6^,^7^ and helical morphologies^2^,^8^, and strong chiral responses are predicted for other geometries^9^,^10^. The chiro-optical properties of nanomaterials originate from the atomic-scale chirality of the inorganic core of NPs^11^, the chiral arrangement of the thiolates on their surfaces^12^, the electronic ‘imprint’ of chirality due to adsorbed chiral organic molecules on NPs surface (for example, DNA and peptides)^13^,^14^, and from the intrinsic chiral geometry of NPs or their assemblies at nano- and submicron-scales^2^,^3^,^6^,^7^,^8^. Currently, chiral nanostructures are prepared using chiral templates, for instance, DNA including the origami approach^2^, helical fibres^15^, twisted nanoribbons^8^ or by lithography^16^, and the primary motivation for the development of chiral nanomaterials is the possibility of creating chiral metamaterials with negative refractive indices^17^. Optical devices utilizing these phenomena are intriguing, but fundamental challenges remain for the practically relevant infrared and visible range.

In this study, we pursue the bioanalytical potential of self-assembled chiral nanoscale superstructures. We demonstrate that the limit of DNA detection reached by side-by-side (SBS) assemblies of Au nanorods (NRs) using chiral bisignate plasmonic signals could be markedly lower than those reported for other widely discussed optical methods employing ultraviolet–visible absorption^18^ of coupled plasmons, fluorescence tagging^19^ and surface-enhanced Raman scattering (SERS)^20^. In addition, these results compete well for single molecular detection of analytes^21^, due to an alternative dependence of optical polarization effects between interdistant plasmonic particles.

## Results

### NR assemblies by PCR

**Figure 1:**
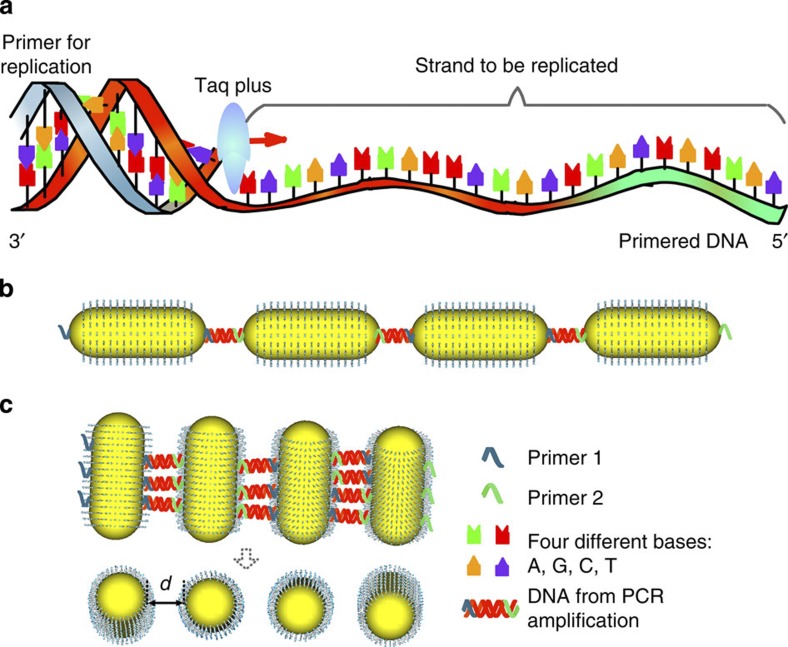
Schematics for PCR assembly of Au NRs. (**a**) PCR replication procedure in which a DNA strand can be ampilified using primer, template DNA, taq plus polymerase and four different DNA bases. (**b**) PCR-based gold NRs ETE assembly. (**c**) PCR-based gold NRs SBS assembly with inter-NR gap *d*; in the bottom part of the panel the DNA chains were removed for clarity.

In this study, chiral assemblies of gold NRs were made using a polymerase chain reaction (PCR) (Fig. 1a)^22^. The use of PCR allowed for the controlled growth of NP and NR assemblies connected by DNA, where the number of thermal cycles determined the lengths and complexity of the resulting superstructures (Fig. 1). The mode of attachment of NRs to each other followed either an end-to-end (ETE) or a SBS assembly pattern, controlled by the placement of the PCR primers (Fig. 1).

**Figure 2:**
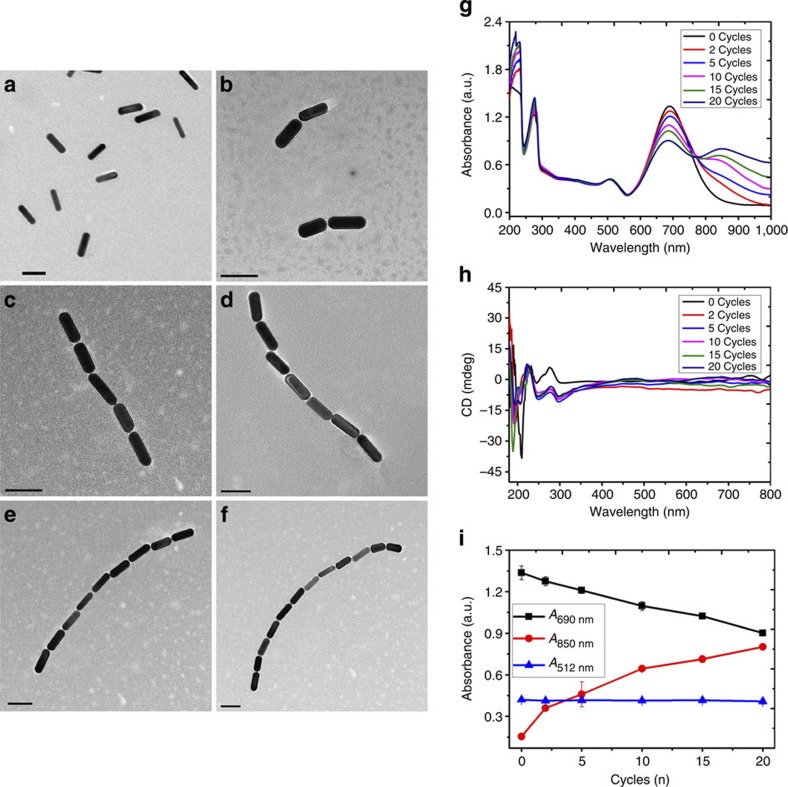
Structure and optical properties of ETE assemblies of Au NRs. (**a**–**f**) Representative TEM images for ETE assembly obtained after different number of PCR cycles, *n=*0 (**a**), 2 (**b**), 5 (**c**), 10 (**d**),15 (**e**) and 20 (**f**); scale bar, 50 nm. (**g**,**h**) Ultraviolet–visible (**g**) and CD spectra (**h**) for ETE assembly obtained for different *n*. (**i**) Intensity of absorption maxima for ETE assemblies obtained for different *n*. The error bars represent the standard deviation of sample measurements.

**Figure 3:**
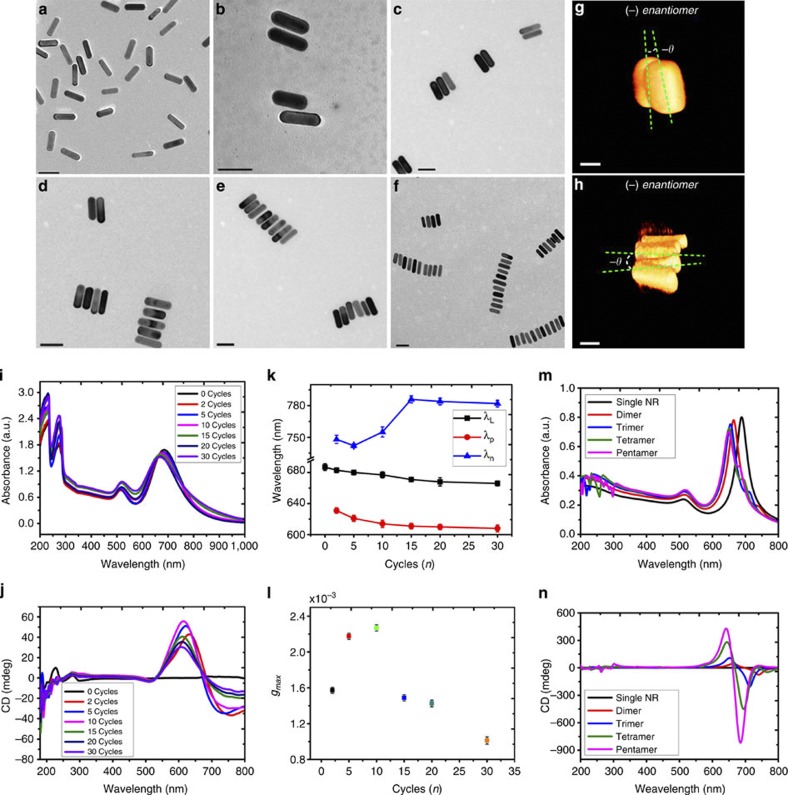
Structure and optical properties of SBS assemblies of Au NRs. (**a**–**f**) Representative TEM images for SBS assemblies obtained after different number of PCR cycles, *n=*0 (**a**), 2 (**b**,**c**), 5 (**d**), 10 (**e**) and 15 (**f**); scale bar, 50 nm. (**g**,**h**) Cryo TEM tomography images for NRs SBS assembled trimer (**g**) and pentamer (**h**); scale bar, 25 nm. (**i**, **j**) Experimental ultraviolet–visible (**i**) and CD spectra (**j**) for SBS assemblies with *n=*0–30. (**k**) Evolution of spectral features of SBS NR assemblies represented by *λ*_L_ (longitudinal peak maximum in ultraviolet–visible spectra), *λ*_p_ (positive peak in plasmonic part of the CD spectra) and *λ*_n_ (negative peak in plasmonic part of the CD spectra) with increasing *n*. (**l**) Dependence of the maximum of chiral anisotropy factor *g*_max_ on *n*. (**m**,**n**) Calculated absorption (**m**) and CD spectra (**n**) for NRs SBS assemblies. The number of NRs, *n,* ranged from 1 to 5. The error bars in **i** and **l** represent the standard deviation of sample measurements.

The gold NRs had lengths and diameters of 62 nm and 22 nm, respectively, with an aspect ratio of 2.9. Preferential binding of thiol-terminated primers to the end facets of the NRs allowed for the ETE growth mode for the NR chains (Fig. 1b)^5^. To obtain SBS assemblies, Au NRs were modified by dithiothreitol (DTT) binding to the end sites and thiol polyethylene glycol. These modifications make NRs stable for a wide range of solution conditions and protects them from excessive modification by thiols^22^. Subsequent addition of the thiolated primer resulted in preferential attachment to the sides of the NRs. Once introduced to the PCR replication system, NRs modified with DNA strands either at their sides or ends acted as ‘monomers’ for the PCR assembly (Fig. 1c) and ‘building blocks’ for the resulting nanoscale assemblies. Variations in the placement of the primers allowed for finely controlled synthesis of extended NR ‘chains’ and ‘ladders’. The number of PCR cycles, *n*, regulated the length of the assemblies (Figs 2a–f and 3a–f, Supplementary Figs S1–S11). The NR attachment patterns were retained until *n*=20 and 30 for ETE and SBS assemblies, respectively (Supplementary Figs S6, S11). Modified Au NRs without PCR (*n*=0) organized sporadically (Figs 2a and 3a). The consistent elongation of NR ‘chains’ and ‘ladders’ with increasing *n* was confirmed by dynamic light scattering measurements of the hydrodynamic diameter (*D*_h_). For the ETE assembly, *D*_h_ increased from 102±2.1 to 701±15 nm as *n* increased from 2 to 20 cycles. For the same values of *n*, *D*_h_ increased from 88±3.1 to 408±23.2 nm in the case of SBS assembly (Supplementary Fig. S12). This change in *D*_h_ correlates well with the increase of statistically averaged number of NRs in ETE and SBS assemblies for different *n* values (Supplementary Figs S13–S17). As expected, the length of ETE is greater than those for SBS assemblies because of the sideway attachment of NRs in the latter case. In concert with previous studies^23^, the transverse plasmon for the ETE assembly changed very little with *n* (Fig. 2), whereas the longitudinal peak shifted to the red (Fig. 2g). For the SBS assemblies (Fig. 3a–f), the longitudinal plasmon band (*λ*_L_) experienced a blue shift by 17 nm from *n=*0 to *n =*30 (Fig. 3i,k).

### Chiral properties of the NR assemblies

The chiroptical properties of the NR assemblies and evaluation of their prospects for bioanalysis were the primary foci of this work. A distinct CD signal was seen in the ultraviolet part of the spectrum from 180 to 250 nm for both ETE and SBS assemblies. This should be attributed to DNA ligands and is expected for this system. A strong bisignate CD wave was seen in the plasmonic *λ*_L_ part of the spectrum between 500 and 800 nm. For the Cotton effect, the 620–800 nm spectral window was negative, whereas for 500–620 nm wavelengths it was positive, which has definitive analogies in molecular systems^24^ although displaying greater CD intensity and *g-*factors (Fig. 3g, Supplementary Fig. S18). Chirality of the assemblies was observed only for SBS and not for ETE assemblies or single DNA-modified NRs (Figs 2h and 3j). The strong chiroplasmonic response was observed for *n* as few as two (Fig. 3j) when assemblies of 2–4 NRs were dominant (Supplementary Fig. S14a). We saw progressively stronger CD signals as *n* increased, indicating that chiral geometries of NR assemblies persisted along with increasing the length of SBS assemblies.

Appearance of the CD signal should be attributed to the twisted structure of the SBS assemblies^22^. The three-dimensional (3D) images of the assemblies obtained with state-of-the-art cryo transmission electron microscopy (TEM) tomography showed a distinct and consistent twist between two adjacent NRs in the SBS assemblies (Fig. 3g,h)^22^. Note these images reflect the conformation of the assemblies as they exist in solution and are not affected by high-vacuum conditions and drying. The negative values of the dihedral angle (*θ*) between the adjacent NRs in SBS assemblies corresponding to the right rotating enantiomers (Supplementary Fig. S16) persist throughout the PCR assembly process^22^. These dihedral angles for dimer, trimer, tetramer and pentamer were consistently negative and equal to −9.0, −7.1, −8.0 and −7.0 degrees, respectively (Fig. 3g,h, Supplementary Figs S16, S17). The preference for one enantiomer as opposed to another is related to symmetry breaking of the parallel NR due to twisting of the connected DNA bridges and the general preference of non-parallel orientation of charged nanoscale rods as the conformation with minimal energy with multiple examples in biomolecules^22^. The consistency of the sign of *θ* leads to strong chirality of SBS-assembled NRs. ETE assemblies do not exhibit CD response (Fig. 2h) because the torsional force of chiral DNA oligomers connecting one end of the rod to another is substantially smaller than in SBS assemblies and therefore the enantiomer distribution is equilibrated. Chirality of the twisted NR assemblies was predicted theoretically^10^ but was never observed experimentally^5^ until recently^22^. Unlike earlier studies by Kadowala and coworkers on chiroplasmonic shifts on chiral coatings^25^, the prospects for analytical and other applications of their chiroplasmonic properties of twisted assemblies in solutions were not considered theoretically or experimentally. We hypothesized it to be a promising research direction due to the intensity of the chiral signals in these assemblies, their bisignate nature, stronger polarization rotation in solution than in thin films and continuous increase of the CD signal for 0<*n<10.* The peak values of the anisotropy factor (*g*-factor) increased from 1.6 × 10^−3^ to 2.3 × 10^−3^ with 2–10 cycles (Fig. 3l, Supplementary Fig. S18).

As the number of PCR cycles increased, both positive (*λ*_p_) and negative (*λ*_n_) CD bands exhibited spectral shifts. The *λ*_p_ CD band moved to the blue part of the spectrum by 24 nm as PCR cycles increased from 2 to 20 cycles (Fig. 3k), whereas *λ*_n_ shifted by 16 nm from 2 to 5 cycles and became broader after 5 cycles. Also important is that as *n* exceeds a certain threshold value, the amount of disorder increases and the CD intensity of the bands stops growing. The larger aggregates do not have the consistency in the signs of their dihedral angles and therefore their CD signals decrease (Fig. 3i). For *n*=20, complex agglomerates formed (Supplementary Fig. S11) and racemization of the assemblies occurred.

The absorbance (Fig. 3n) and CD (Fig. 3m) spectra calculated for SBS assemblies of 2–5 NRs showed excellent agreement with experimental results. The simulated electric fields on gold surface (*E*-fields) under right/left circular polarized (RCP/LCP) light excitation at *λ*_p_ and *λ*_n_ bands showed that the twisted SBS assemblies were indeed chiral: the coupling efficiencies to RCP and LCP (Supplementary Fig. S19) differ considerably. Agreeing with experimental results, the ETE-assembled structure did not show any CD signal (Fig. 2h) in the plasmonic region. The simulations of ETE NR trimer performed according to the 3D geometry-based state-of-the-art cryo TEM tomography and indeed exhibited a very weak CD signal (Supplementary Fig. S20).

### NR assemblies for attomolar DNA biosensing

Chirality of SBS assemblies employing different reactant DNA templates can be potentially used for biosensing of oligonucleotides. The possibility of improving the limit of detection (LOD) by taking advantage of the bisignate wave-shape of the CD signals can be one of the advantages of the method. As *n* increases, *λ*_p_ becomes more positive, whereas *λ*_n_ becomes more negative. These spectroscopic changes occur in synch with each other, which essentially increases the detected signal and improves the signal-to-noise ratio. Note also that nanoscale assemblies amplify the chiral adsorption compared with the atomic-scale chirality in organic molecules. In addition to amplification of signal due to bisignate nature of the CD wave and nanoscale dimentions of chiral chromophores, there are also other factors related to dependence of intensity of CD signal on inter-NR gap, *d*. This dependence is conducive to ultrasensitive detection of biomacromolecules and represents one of the key advantages of this methods compared to others based on plasmon coupling.

**Figure 4:**
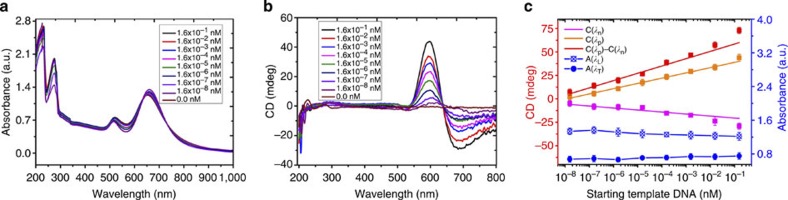
DNA analysis with SBS NRs assemblies. (**a**,**b**) Experimental ultraviolet–visible (**a**) and CD spectra (**b**) for NR assemblies obtained for different DNA concentrations starting from 0.156 nM with stepwise 10 × dilution. (**c**) Calibration curves obtained using CD and ultraviolet–visible spectra of SBS assemblies. The error bars represent the standard deviation of sample measurements.

We evaluated CD and other spectral optical responses for different amounts of target DNA oligomers. The calculated LOD was found to be 3.9 aM, 8.1 aM and 3.7 aM based on CD intensity of C(*λ*_p_), C(*λ*_n_) and C(*λ*_p_)–C(*λ*_n_), respectively (Fig. 4). The LOD values above characterizes this techniques as substantially more sensitive than typical PCR methods with or without NPs that give LOD=0.1 fM (ref. 26). To validate the bioanalysis by chiroplasmonic effects, the widely used dilution method (see Methods) with *a priori* known concentrations of DNA was adopted (Supplementary Fig. S21). The concentrations returned by the chiroplasmonic method matched those expected for the specific dilution. The uncertainty coefficient, *u*, was as low as 0.0367 (see Methods) and indicative of the high accuracy of the method. Such *u* is associated, among other factors, With PCR amplification, high *g*, and improved signal-to-noise ratio due to bisignate nature of the chiroplasmonic spectra.

The use of CD spectroscopy is not uncommon for biosensing and sometimes offer better levels of detection compared with ultraviolet–visible^18^, fluorescence spectroscopy^27^, and electrochemistry-based methods^28^, with the best LODs equal to 10,200 and 500 fM, respectively, especially in combination with plasmonic substrates^29^,^30^. However it never reached the level of attomolar (10^−18^ M) level of detection. The femtomolar detection level (10^−15^ M) is not sufficient to meet the demands of biomedical and environmental applications for a variety of clinical tasks especially for reliable early diagnosis of diseases using DNA biomarkers in complex biological samples.

It is relevant to compare analytical capabilities of chiroplasmonic method with its parent/related techniques, for instance, PCR and SERS that do not use CD spectra modulated by plasmonic particles. In some cases methods based on polaron coupling were able to reach nearly single-molecule detection capabilities^20^ and high clinical relevance for detection of prostate cancer^21^. We compared chiroplasmonic method with well-established and highly sensitive reverse transcription PCR (RT-PCR) used for clinical and forensic purposes. LOD for the same DNA strands as in Fig. 4 was found to be 156 aM for RT-PCR (Supplementary Fig. S22). The NR assembly method was, therefore, *ca.* 40 times more sensitive than RT-PCR. The parallel experiments with chiroplasmonic method yielded identical concentrations with the chiroplasmonic bioanalysis.

**Figure 5:**
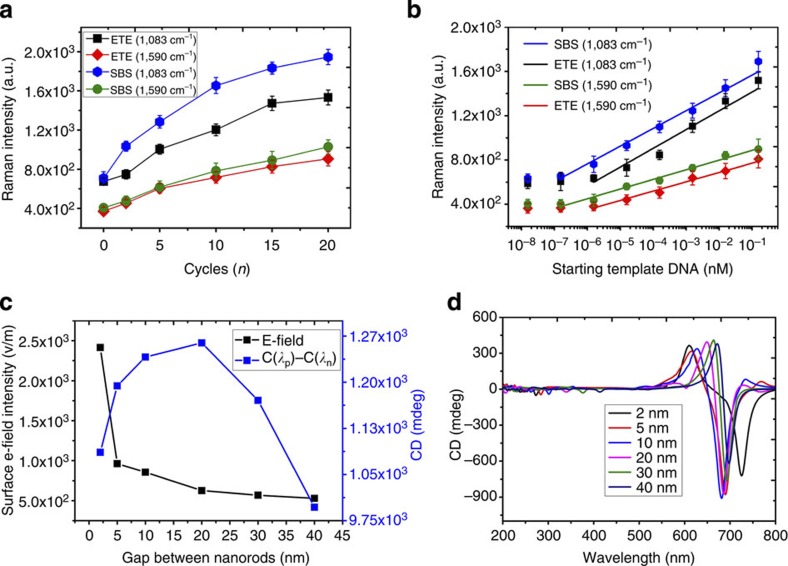
DNA analysis using SERS capabilities. (**a**,**b**) SERS intensity of 4-ATP tag at 1,083 cm^−1^ and 1,590 cm^−1^ for ETE and SBS NR assemblies obtained after different *n* (**a**) and different starting DNA concentrations (**b**). (**c**) Calculated dependence of the intensity of the surface *E*-field and CD intensity for a NR pentamers with gaps of 2, 5, 10, 20, 30 and 40 nm. (**d**) Calculated CD spectra for NR pentamers with gaps of 2, 5, 10, 20, 30 and 40 nm. The error bars represent the standard deviation of sample measurements.

Analytical capabilities of SERS are being rapidly advanced in many laboratories including successful SERS analysis using ETE and SBS assemblies of Au NRs^23^,^31^ featuring high-intensity *E*-fields between the rods, and therefore, deserve special attention in this study. One of the best case scenarios for DNA detection would probably be the use a high-intensity SERS tag, such as 4-aminothiophenol (4-ATP) with Raman-active transitions *υ*(C–S) at 1,083 cm^−1^ and *υ*(C–C) at 1,590 cm^−1^. Its SERS signal is further enhanced by *E-*fields on gold surface amplified after bridging of NRs by DNA. The Raman intensity of 4-ATP increased with increasing *n* for ETE assemblies with addition of new NRs (Fig. 5a, Supplementary Figs S23–S25). Note that Lee *et al.*^31^ observed a continuous decrease of SERS and *E-*field intensity with increasing numbers of NRs in SBS assemblies, whereas CD signal in DNA-bridged ETE assemblies display the opposite trend and increase for *n<*10 which contributes to improved sensitivity of the chiroplasmonic method. LOD using the strongest SERS line of 4-ATP at 1,083 cm^−1^ was 1.14 and 1.58 fM for SBS and ETE assemblies (Fig. 5b), respectively, which is 290 and 403 times higher than for chiroplasmonic method for the identical DNA strand. Importantly, detection of long DNA is inherently suboptimal using SERS because formation of hotspots critical for its success requires the gaps between NRs to be small, that is, *ca.* 0.5–2 nm. The same is also true for SERS detection of all high molecular weight compounds that are larger than the size of optimal hotspots and similar spectroscopic methods based on strong polaron coupling, such as surface-enhanced Raman optical activity^24^. Molecules with diameters >2 nm are particularly common in bioanalysis. As indicated by the calculations in Fig. 5c,d, the read-out parameter used in chiroplasmonic method, C(*λ*_p_)–C(*λ*_n_), is much less sensitive to the distance between the NRs than the surface *E*-field necessary to enhance scattering from 4-ATP or other Raman tags. In fact, it initially increases for DNA-relevant size regime rather than decreasing as *E-*field intensity. Polarization rotation reaches maximum for the NR gap of *ca.* 20 nm, whereas the intensity of the surface *E-*field generated by NRs drops off dramatically when gap reaches 5 nm (Fig. 5c, Supplementary Fig. S26).

## Discussion

We showed that the SBS assemblies of plasmonic NRs with strong polarization rotation make possible detection of DNA markers with unusually low LOD that is greatly needed for medical diagnostics, forensics and environmental needs. The physical phenomena behind this capability include enhancement of polarization rotation by plasmonic structures, chiral symmetry breaking for SBS assemblies and bisignate nature of CD spectra. Chiroplasmonic method of detection has sensitivity advantage for the analysis of biomolecules >2 nm, while other plasmonic methods could be preferred for smaller analytes, unless steps for narrowing the gap by depositing additional layers of plasmonic material are taken. In perspective, the high sensitivity of the CD signal to geometry of the twisted NR assembly allows for experimental observation of the torsional dynamics of helical systems in solutions and better understanding of the 3D geometry of plasmonic assemblies.The strong polarization rotation in DNA-bridged SBS assemblies can also be utilized in intracellular monitoring of low-occurrence markers and signaling molecules.^39^

## Methods

### Au nanorod preparation

Au NRs were synthesized by Au seeds growth method^32^. Synthesis of Au seeds: hydrogen tetrachloroaurate trihydrate (HAuCl_4_·3H_2_O) was dissolved in 2.5 ml, 0.2 M hexadecyltrimethylammonium bromide (CTAB) solution, added by 0.3 ml pre-cooled 300 μl, 0.01 M sodium borohydride (NaBH_4_) and quickly mixed for 2 min and left to reaction at 25 °C for 2 h. The growth of Au NR, 0.15 ml of 0.004 M AgNO_3_ (NR): 70 μl of 0.079 M (ascorbic acid, Vc) was added to 5 ml, 0.001 M, HAuCl_4_ was added to 5 ml, 0.2 M CTAB solution and left to reduce for 2 min, finally 12 μl of prepared Au seeds were added, strongly stirred for 20 s and left at 25 °C. The concentration of gold NRs was 0.25 nM.

### NR modification

Modification of Au NRs included primer on end, side and 4-ATP modifications.

### End modification

A 1 ml aliquot of synthesized Au NRs was first centrifuged (10,000 *g*, 10 min). The precipitate was then dissolved in 200 μl of 0.005 M CTAB solution (five times concentrated). The Au NRs modification of the primer was carried out at 25 °C for 12 h with a reaction ratio of 80:1 between the Au NRs and the primer. Then unreacted primer was removed by centrifuging (7,000 *g*, 10 min) and dissolved in 200 μl of 0.005 M CTAB solution.

### Side modification

A 1 ml aliquot of synthesized Au NRs was five times concentrated into 200 μl of 0.005 M CTAB solution by centrifugation. Then the end sites were modified with DTT for 8 h with a molar ratio of 10:1 between the Au NRs and the primer. The DTT-modified Au NRs were centrifuged again (7,000 *g*, 10 min) and dissolved in 200 μl of 0.005 M CTAB solution. Then the Au NRs were modified by the addition of polyethylene glycol with a molar ratio of 120:1. Finally, the side modification of the primer was reacted for 10 h at a molar ratio of 400:1 between the primers and the Au NRs. The Au NRs were then centrifuged (70,000 *g*, 10 min) and stored in 200 μl of 0.005 M CTAB solution.

### SERS tag modification

For SERS measurement, 4-ATP was used to modify the Au NR after being modified by primers. The 4-ATP was modified after the primer was attached with the same modification ratio for end and side modifications. Generally, 4-ATP modification employed similar procedures with the same reaction ratio as that of the NRs modified with primer. The molar ratio between the Au NRs and the 4-ATP was set at 400:1 and reacted for 8 h both for the NRs used for ETE and SBS assemblies. The Au NRs were then centrifuged at 70,000 r.p.m. for 10 min and finally dispersed in 200 μl of 0.005 M CTAB solution. After 4-ATP modification, the as-prepared NRs were employed in PCR-based assemblies with different cycles and the assembled products were monitored in the liquid phase.

### PCR conditions and Au NR assembly

Using Lambda DNA as a template^23^, the PCR reaction was performed in a 100 μl amplification system. The reaction mixture contained 10 μl of PCR buffer, 2 μl of dNTPs (1 mM), 1 μl of template DNA, 1 μl (5 U) of Taq Plus polymerase and 10 μl of each Au NR-forward/reverse-primer (F/R-primer) conjugates (Au NR-F/R-primer); finally, amplification mixture was set to 100 μl by adding 66 μl of ultrapure water. The 5 × PCR buffer contained of 50 M KCl, 10 mM tris–HCl, pH 9.0 at 25 °C, 0.1% TritonX-100 and 1.5 mM MgCl_2_. Each of the Au NR-F/R-primer conjugates and Au NR-reverse-primer (R-primer) conjugates were concentrated to 1.25 nmol. The thermal cycling protocol began with a 3 min predenaturation step at 94 °C, followed by varying cycles of 94 °C denaturation (30 s), 60 °C annealing (30 s) and 72 °C extension (1 min) steps, followed by a further extension for 10 min at 72 °C. Last, the PCR system was held at 4 °C for *ca.* 10 min before use. Sequences for F-primer and R-primer were listed as follows: F-primer: TGGCTGACCCTGATGAGTTCG; R-primer: GGGCCATGATTACGCCAGTT. The assembly parameter of PCR cycle (*n*) and starting template DNA was set as follows: the *n* was set as 0, 2, 5, 10, 15, 20 and 30 cycles with starting template DNA concentration of 0.156 nM. The template DNA concentration was set by 10 times stepwise dilution of the starting material, 0.156 nM to 0.0156, fM.

### Analytical calculations, experimental statistics and uncertainty analysis

The LOD was calculated according to the high sensitivity analysis. The calibration curve was plotted as


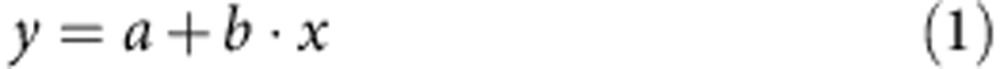


where *a* and *b* are the variable obtained via least-square root linear regression for the signal–concentration curve for variable *y* representing the CD signal (mdeg) at DNA concentration of *x* (nM).

When


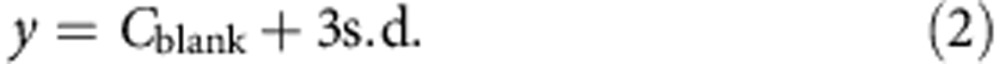


where s.d. is the standard deviation and *C*_blank_ is the CD of signal of blank sample (without DNA).

The LOD was calculated as follows:


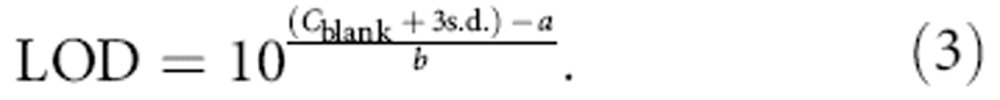


The uncertainty for LOD of our assay was calculated using statistical analysis methods based on standard deviations of different concentration points (A-type uncertainty). The standard deviation (s.d.) was calculated according to the well-known formula:


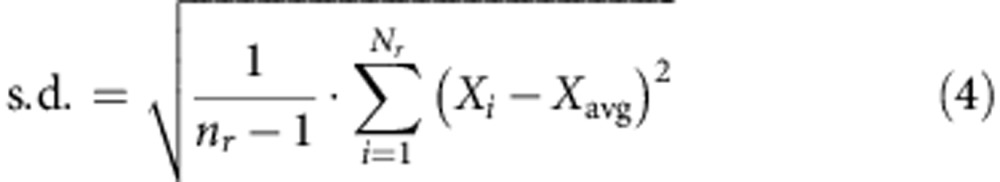


where *n*_*r*_ is the total number of the samples. *X*_*i*_ is the *i*th sample of the series of measurements. *X*_avg_ is the average value of the CD (or other) signals obtained for the specific series of identical samples repeated *n*_*r*_ times.

The uncertainty coefficient, *u* was calculated as follows:


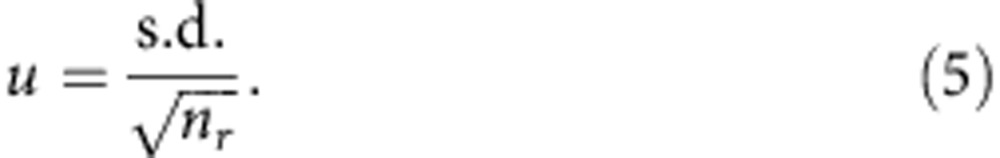


The sample repeat number, *n*_*r*_, was *n*_*r*_=9 for all of the experiments. For DNA detection following the chiroplasmonic protocol presented below, the uncertainty coefficient was 0.0367.

According to the methods of uncertainty analysis accepted in analytical chemistry^33^, this value of uncertainty coefficient corresponds to a high accuracy method.

### Calibration protocol

Progressive dilution method with known concentrations of analytes is used for calibration of a wide variety of analytical methods including those for exceptionally small concentrations and single-molecule detection limits. These methods can be based on ultraviolet–visible^18^, fluorescence spectroscopy^27^, or electrochemistry^28^. The reliability of this calibration technique stems from the high accuracy of volume measurements when a sample of known (high) concentration is diluted according to the power law.

The template DNA concentrations was acquired by stepwise 10 × dilution, following the procedure accepted in the field of bioanalysis and other branches of analytical chemistry^18^,^34^. For each dilution, 5 μl DNA solution (by pipette with 1–10 μl measurement range) was added to 45 μl (by pipette with 5–50 μl measurement range) ultra-pure water. The pipette tips were replaced and discarded every time. Analogous procedure was carried out for the blank experiment without starting DNA analyte and was used in equation (2) as *C*_blank_. One microlitre of as-diluted DNA solution for a specific concentration was added into 100 μl PCR system for amplification and subsequent analysis by CD spectroscopy.

### RT-PCR detection protocol

The RT-PCR was performed by standard procedure according to the process described by Ma *et al.*^35^ RT-PCR was carried out on CFX-96 real-time PCR system with 25 ml amplification volume. The PCR amplification mixture was composed of 1.5 ml 20 × EvaGreen dye, 2.5 ml of 1 mM dNTP, 2.5 ml of 10 × PCR buffer, 0.25 ml of 5 U Taq DNA polymerase and 0.5 ml of 2.5 mM upstream and downstream primer, respectively, and finally ultra-pure water was added to have a volume of 25 ml. The PCR cycling parameters were set as 94 °C denaturation (30 s), 60 °C annealing (30 s) and 72 °C extension (1 min) steps, followed by a further extension for 10 min at 72 °C. Fluorescence measurements were taken after each annealing step. The standard curve for RT-PCR is plotted in Supplementary Fig. S22 according to the number of the threshold cycle, *n*_threshold_, defined as the number of PCR cycles when the fluorescence reached the value of 900 (a.u.) for specific concentration of DNA (exponential amplification stage).

### Computer simulations

Computer simulations ETE and SBS assemblies were performed using CST Microwave Studio^36^,^37^. The geometry of the NR ladders and chains was defined by *d*, surface-to-surface gap between Au NRs, the angle between the long axes of the two NRs. The propagation of excitation beam was defined by *φ*_*x*_ and *φ*_*z*_, the angles between excitation beam and *x* axis, *z* axis, respectively. The surface of gold NR is composed of two semispherical surfaces and one side cylinder surface. Surface *E*-field simulations by RCP and LCP were carried out at *λ*p and *λ*n, respectively. Surface *E*-field enhancement simulations were carried out using linearly polarized beam with *E*-field vector parallel and perpendicular with longitudinal direction of NR. The total *E*-field enhancement was the sum of these two fields. Simulations of CD and absorbance spectra were accomplished by parameter sweep of *φ*_*z*_ and *φ*_*x*_ from 0 to 2*π* with step of *π/*6 (30°), which was similar to theoretical methods previously explored^9^,^14^,^38^.

### Instrumentation

The ultraviolet–visible spectra were measured using a ultraviolet–visible spectrometer (200–1,000 nm) in a quartz cell. The CD spectra were performed on a Bio-Logic MOS-450 CD spectrometer. TEM micrographs were collected on a JEOL-2010 microscope operated at 120 kV. The 3D reconstruction of electron tomography was carried out using a Tecnai Spirit 120 kV TEM. Dynamic light scattering data were obtained using a Malvern Zetasizer ZS instrument with a 632.8 nm laser source and a backscattering detector at 173°. Raman scattering spectra were measured in liquid cell using a LabRam-HR800 Micro-Raman spectrometer with Lab-spec 5.0 software. The slit and pinhole were set at 100 and 400 mm, respectively, an air-cooled He-Ne laser for 632.8 nm excitation with a power of ~8 mW.

## Author contributions

C.X., L.W. and N.A.K. designed the experiments, interpreted and analyzed the data, conceptualized the findings and co-wrote the paper. W.M., H.K., L.X., L.D. performed the experiments, characterizations, prepared the samples and carried out computer calculations. W.M. and H.K. analyzed the data and carried out three-dimensional reconstruction of electron tomography.

## Additional information

**How to cite this article:** Ma, W. *et al.* Attomolar DNA detection with chiral nanorod assemblies. *Nat. Commun.* 4:2689 doi: 10.1038/ncomms3689 (2013).

## Supplementary Material

Supplementary Information Supplementary Figures S1-S26

## References

[b1] Sheikholeslami S., Jun Y. W., Jain P. K. & Alivisatos A. P. Coupling of optical resonances in a compositionally asymmetric plasmonic nanoparticle dimer. Nano Lett. 10, 2655–2660 (2010). 20536212 10.1021/nl101380f

[b2] Kuzyk A. *et al.* DNA-based self-assembly of chiral plasmonic nanostructures with tailored optical response. Nature 483, 311–314 (2012). 22422265 10.1038/nature10889

[b3] Chen W. *et al.* Nanoparticle superstructures made by polymerase chain reaction: collective interactions of nanoparticles and a new principle for chiral materials. Nano Lett. 9, 2153–2159 (2009). 19320495 10.1021/nl900726s

[b4] Xia Y. H., Zhou Y. L. & Tang Z. Y. Chiral inorganic nanoparticles: origin, optical properties and bioapplications. Nanoscale 3, 1374–1382 (2011). 21301709 10.1039/c0nr00903b

[b5] Nie Z., Petukhova A. & Kumacheva E. Properties and emerging applications of self-assembled structures made from inorganic nanoparticles. Nat. Nanotech. 5, 15–25 (2010). 10.1038/nnano.2009.453 20032986

[b6] Mastroianni A. J., Claridge S. A. & Alivisatos A. P. Pyramidal and chiral groupings of gold nanocrystals assembled using DNA scaffolds. J. Am. Chem. Soc. 131, 8455–8459 (2009). 19331419 10.1021/ja808570g PMC2767249

[b7] Yan W. J. *et al.* Self-assembly of chiral nanoparticle pyramids with strong R/S optical activity. J. Am. Chem. Soc. 134, 15114–15121 (2012). 22900978 10.1021/ja3066336

[b8] Srivastava S. *et al.* Light-controlled self-assembly of semiconductor nanoparticles into twisted ribbons. Science 327, 1355–1359 (2010). 20150443 10.1126/science.1177218

[b9] Govorov A. O., Fan Z., Hernandez P., Slocik J. M. & Naik R. R. Theory of circular dichroism of nanomaterials comprising chiral molecules and nanocrystals: plasmon enhancement, dipole interactions, and dielectric effects. Nano Lett. 10, 1374–1382 (2010). 20184381 10.1021/nl100010v

[b10] Auguié B., Alonso-Gómez J. L., Guerrero-Martínez A. s. & Liz-Marzán L. M. Fingers crossed: optical activity of a chiral dimer of plasmonic nanorods. J. Phys. Chem. Lett. 2, 846–851 (2011). 10.1021/jz200279x 26295617

[b11] Dolamic I., Knoppe S., Dass A. & Burgi T. First enantioseparation and circular dichroism spectra of Au-38 clusters protected by achiral ligands. Nat. Commun. 3, 798 (2012). 22531183 10.1038/ncomms1802 PMC3337976

[b12] Bovet N., McMillan N., Gadegaard N. & Kadodwala M. Supramolecular assembly facilitating adsorbate-induced chiral electronic states in a metal surface. J. Phys. Chem. B 111, 10005–10011 (2007). 17661515 10.1021/jp074056s

[b13] Lu F. *et al.* Discrete nanocubes as plasmonic reporters of molecular chirality. Nano Lett. 13, 3145–3151 (2013). 10.1021/nl401107g 23777419

[b14] Slocik J. M., Govorov A. O. & Naik R. R. Plasmonic circular dichroism of peptide-functionalized gold nanoparticles. Nano Lett. 11, 701–705 (2011). 21207969 10.1021/nl1038242

[b15] Guerrero-Martínez A., Alonso-Gómez J. L., Auguié B., Cid M. M. & Liz-Marzán L. M. From individual to collective chirality in metal nanoparticles. Nano Today 6, 381–400 (2011).

[b16] Hentschel M., Schäferling M., Weiss T., Liu N. & Giessen H. Three-dimensional chiral plasmonic oligomers. Nano Lett. 12, 2542–2547 (2012). 22458608 10.1021/nl300769x

[b17] Soukoulis C. M., Linden S. & Wegener M. Negative refractive index at optical wavelengths. Science 315, 47–49 (2007). 17204630 10.1126/science.1136481

[b18] Elghanian R., Storhoff J. J., Mucic R. C., Letsinger R. L. & Mirkin C. A. Selective colorimetric detection of polynucleotides based on the distance-dependent optical properties of gold nanoparticles. Science 277, 1078–1081 (1997). 9262471 10.1126/science.277.5329.1078

[b19] Zhang Z., Sharon E., Freeman R., Liu X. & Willner I. Fluorescence detection of DNA, adenosine-5′-triphosphate (ATP), and telomerase activity by zinc(II)-protoporphyrin IX/G-quadruplex labels. Anal. Chem. 84, 4789–4797 (2012). 22540661 10.1021/ac300348v

[b20] Lim D.-K., Jeon K.-S., Kim H. M., Nam J.-M. & Suh Y. D. Nanogap-engineerable Raman-active nanodumbbells for single-molecule detection. Nat. Mater. 9, 60–67 (2010). 20010829 10.1038/nmat2596

[b21] Rodríguez-Lorenzo L., de la Rica R., Álvarez-Puebla R. A., Liz-Marzán L. M. & Stevens M. M. Plasmonic nanosensors with inverse sensitivity by means of enzyme-guided crystal growth. Nat. Mater. 11, 604–607 (2012). 22635043 10.1038/nmat3337

[b22] Ma W. *et al.* Chiral plasmonics of self-assembled nanorod dimers. Sci. Rep. 3, 1934 (2013). 23752317 10.1038/srep01934 PMC3678134

[b23] Lee A. *et al.* Probing dynamic generation of hot-spots in self-assembled chains of gold nanorods by surface-enhanced raman scattering. J. Am. Chem. Soc. 133, 7563–7570 (2011). 21513327 10.1021/ja2015179

[b24] Berova N., Bari L. D. & Pescitelli G. Application of electronic circular dichroism in configurational and conformational analysis of organic compounds. Chem. Soc. Rev. 36, 914–931 (2007). 17534478 10.1039/b515476f

[b25] Mulligan A. *et al.* Going beyond the physical: instilling chirality onto the electronic structure of a metal. Angew. Chem. 117, 1864–1867 (2005). 10.1002/anie.200462265 15714452

[b26] Deng H. *et al.* Gold nanoparticles with asymmetric polymerase chain reaction for colorimetric detection of DNA sequence. Anal. Chem. 84, 1253–1258 (2012). 22243128 10.1021/ac201713t

[b27] Cui D. *et al.* Self-assembly of quantum dots and carbon nanotubes for ultrasensitive DNA and antigen detection. Anal. Chem. 80, 7996–8001 (2008). 18816142 10.1021/ac800992m

[b28] Park S. J., Taton T. A. & Mirkin C. A. Array-based electrical detection of DNA with nanoparticle probes. Science 295, 1503–1506 (2002). 11859188 10.1126/science.1067003

[b29] Kravets V. G. *et al.* Singular phase nano-optics in plasmonic metamaterials for label-free single-molecule detection. Nat. Mater. 12, 304–309 (2013). 23314104 10.1038/nmat3537

[b30] Hendry E. *et al.* Ultrasensitive detection and characterization of biomolecules using superchiral fields. Nat. Nanotech. 5, 783–787 (2010). 10.1038/nnano.2010.209 21037572

[b31] Lee A. *et al.* Side-by-side assembly of gold nanorods reduces ensemble-averaged SERS intensity. J. Phys. Chem. C. 116, 5538–5545 (2012).

[b32] Nikoobakht B. & El-Sayed M. A. Preparation and growth mechanism of gold nanorods (NRs) using seed-mediated growth method. Chem. Mater. 15, 1957–1962 (2003).

[b33] Ellison S. L. R. (Eds) A.W. Eurachem/CITAC guide: Quantifying Uncertainty in Analytical Measurement, Analytical Measurement, Third edition (2012).

[b34] Ma W. *et al.* A PCR based magnetic assembled sensor for ultrasensitive DNA detection. Chem. Commun. 49, 5369–5371 (2013). 10.1039/c3cc41674g 23661252

[b35] Wang L. *et al.* Side-by-Side and End-to-End Gold Nanorod Assemblies for Environmental Toxin Sensing. Angew. Chemie Int. Ed., 26, 5472–5475 (2010). 10.1002/anie.200907357 20602373

[b36] Alvarez-Puebla R. A. *et al.* Gold nanorods 3D-supercrystals as surface enhanced Raman scattering spectroscopy substrates for the rapid detection of scrambled prions. Proc. Natl Acad. Sci. USA 108, 8157–8161 (2011). 21536908 10.1073/pnas.1016530108 PMC3100977

[b37] Lilly G. D., Agarwal A., Srivastava S. & Kotov N. A. Helical assemblies of gold nanoparticles. Small 7, 2004–2009 (2011). 21695784 10.1002/smll.201100536

[b38] Zhukovsky S. V., Kremers C. & Chigrin D. N. Plasmonic rod dimers as elementary planar chiral meta-atoms. Opt. Lett. 36, 2278–2280 (2011). 21685992 10.1364/OL.36.002278

[b39] Xu L. *et al.* Regiospecific Plasmonic Assemblies for *in-situ* Raman Spectroscopy in Live Cells. J. Am. Chem. Soc. 134, 1699–1709 (2012). 22192084 10.1021/ja2088713 PMC3277787

