# Supplementary material for: Attomolar DNA detection with chiral nanorod assemblies
Source: Nat Commun. 2013 Oct 28;4:2689. doi: 10.1038/ncomms3689 (PMC3826651; doi:10.1038/ncomms3689)
Supplement: Supplementary Information — Supplementary Figures S1-S26 [file ncomms3689-s1.pdf]

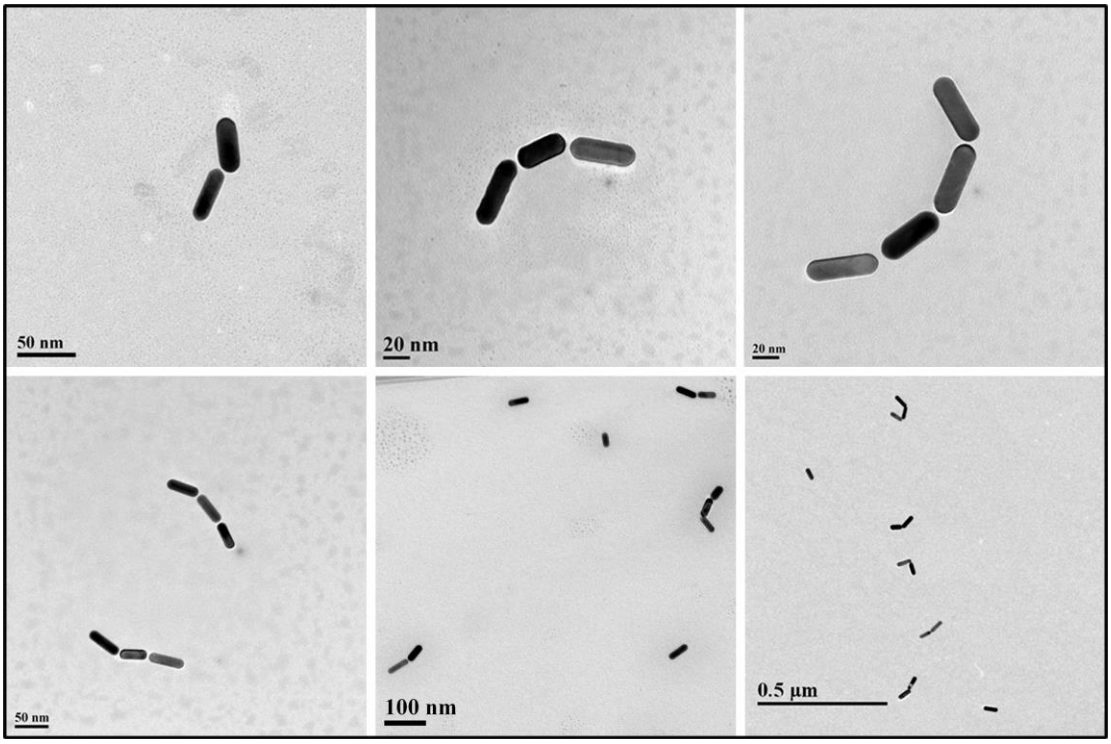

Supplementary Figure S1. Representative TEM images for ETE assembly after 2 PCR cycles.

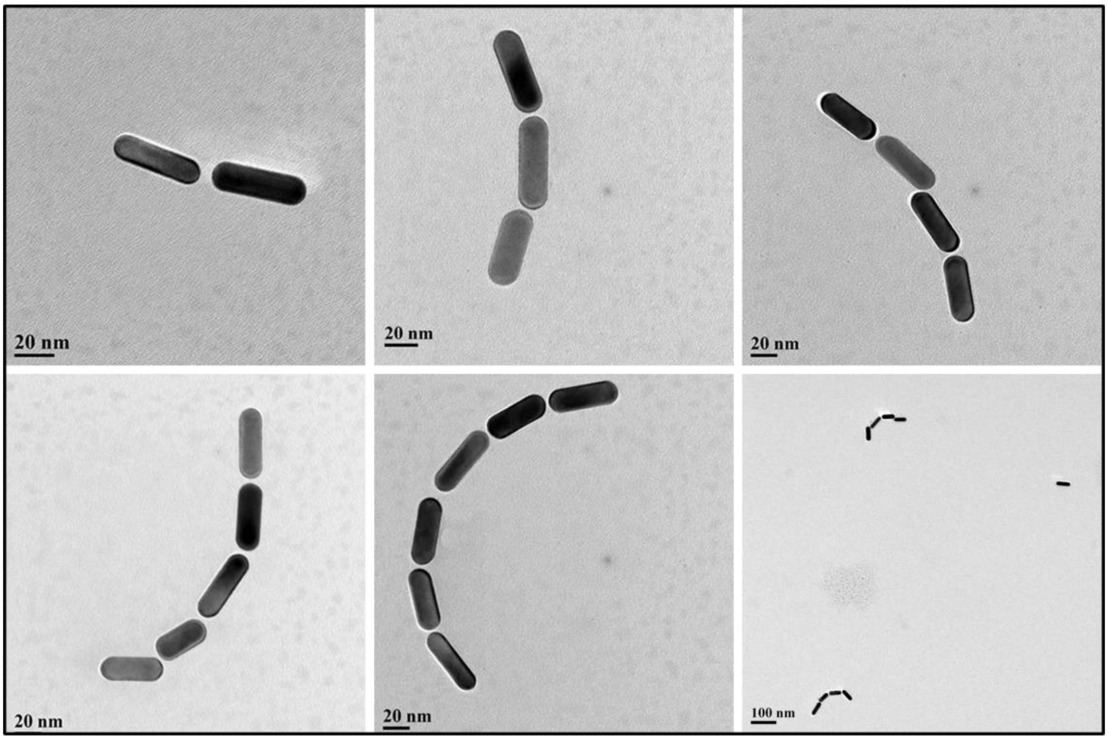

Supplementary Figure S2. Representative TEM images for ETE assembly after 5 PCR cycles.

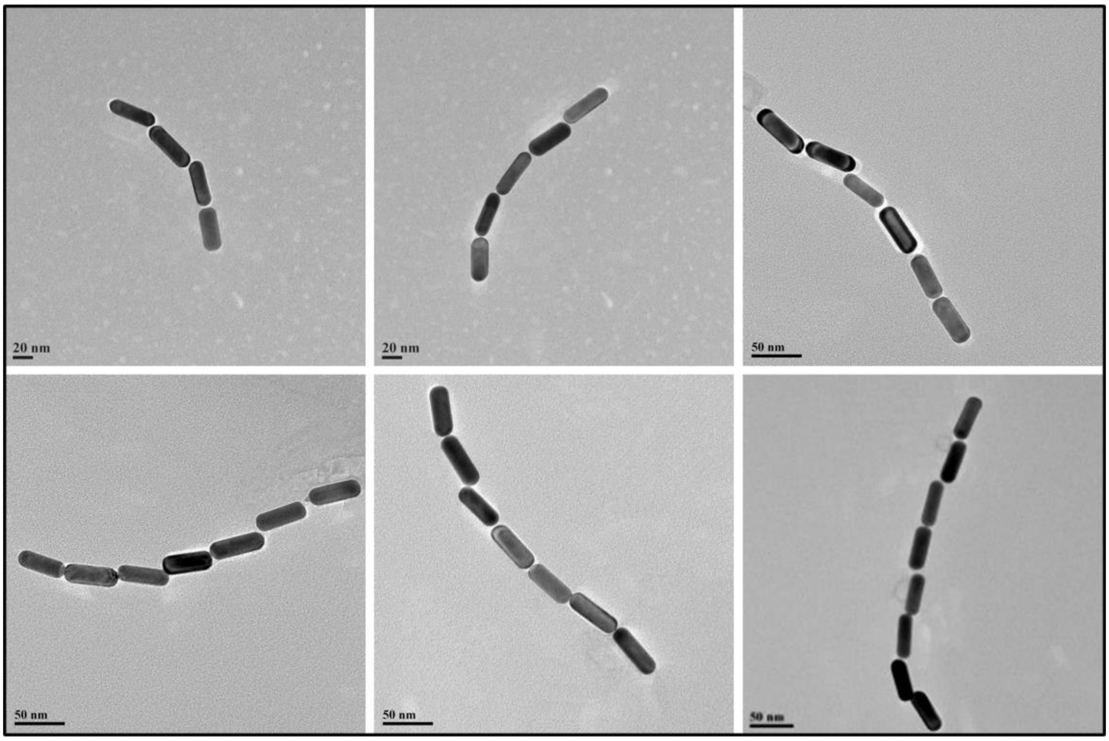

Supplementary Figure S3. Representative TEM images for ETE assembly after 10 PCR cycles.

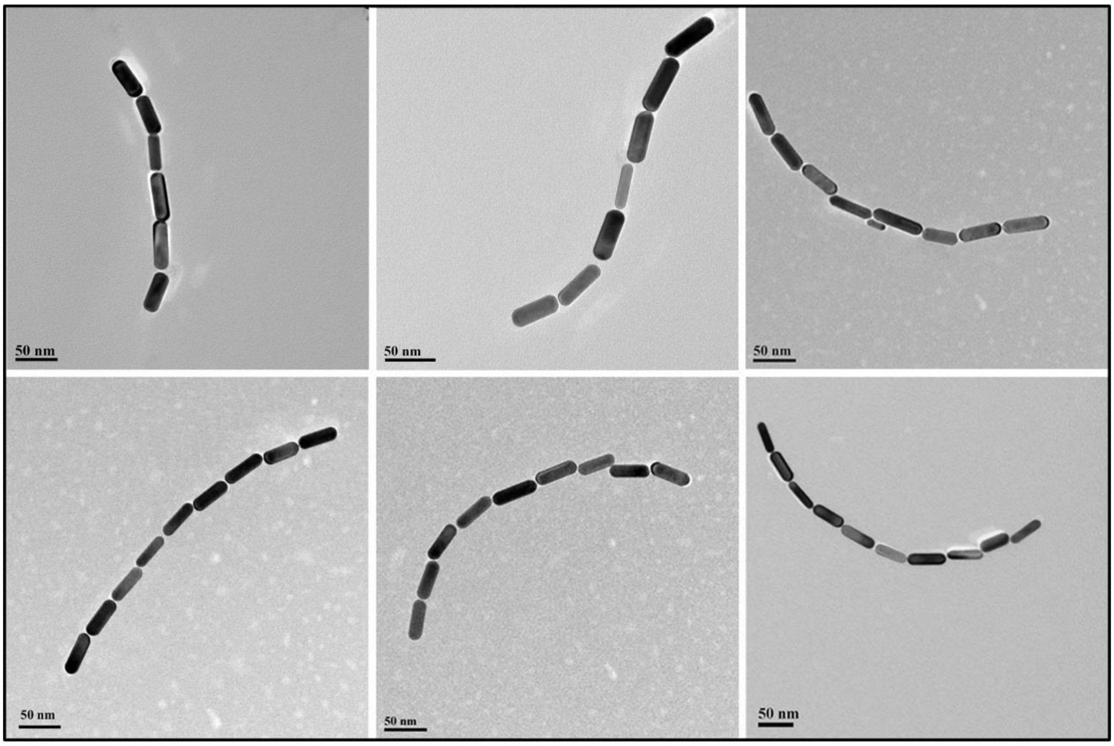

Supplementary Figure S4. Representative TEM images for ETE assembly after 15 PCR cycles.

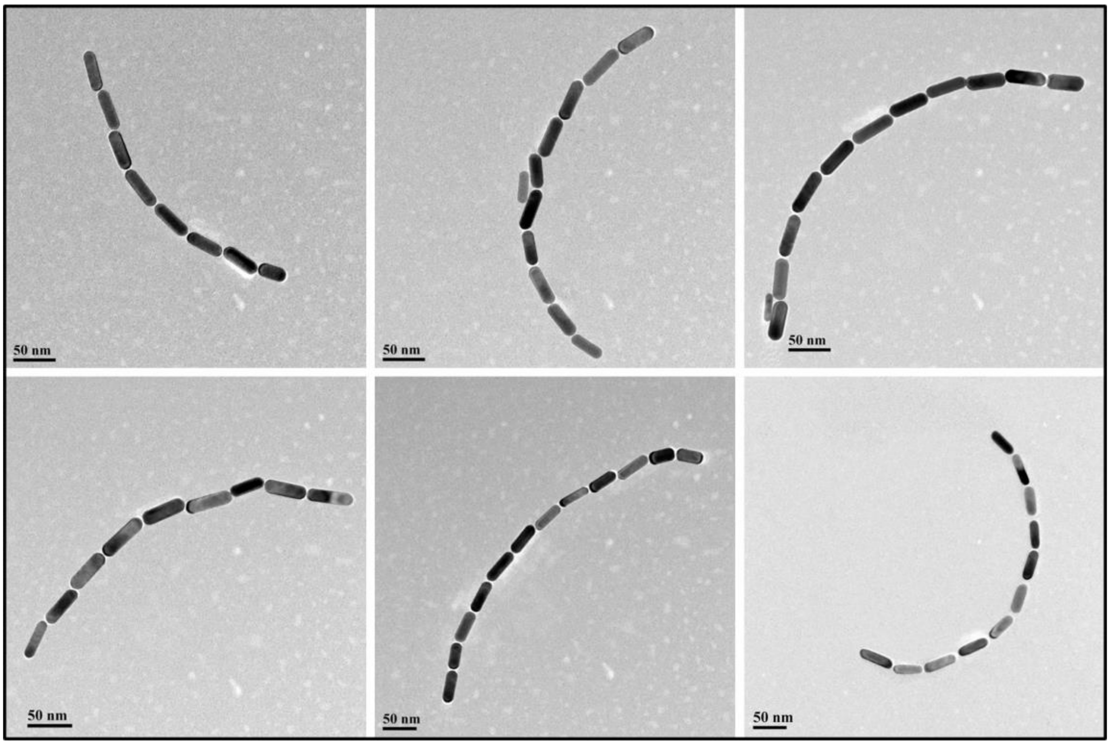

Supplementary Figure S5. Representative TEM images for end to end assembly after 20 PCR cycles.

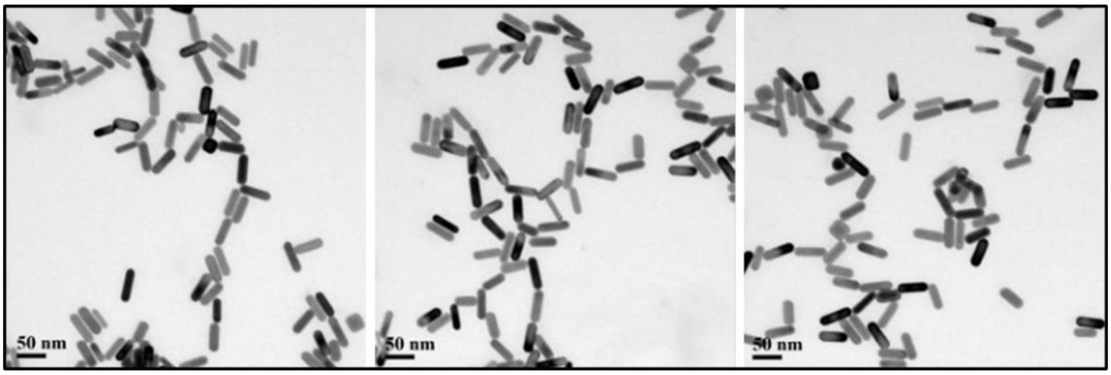

Supplementary Figure S6. Representative TEM images for ETE assembly after 30 PCR cycles.

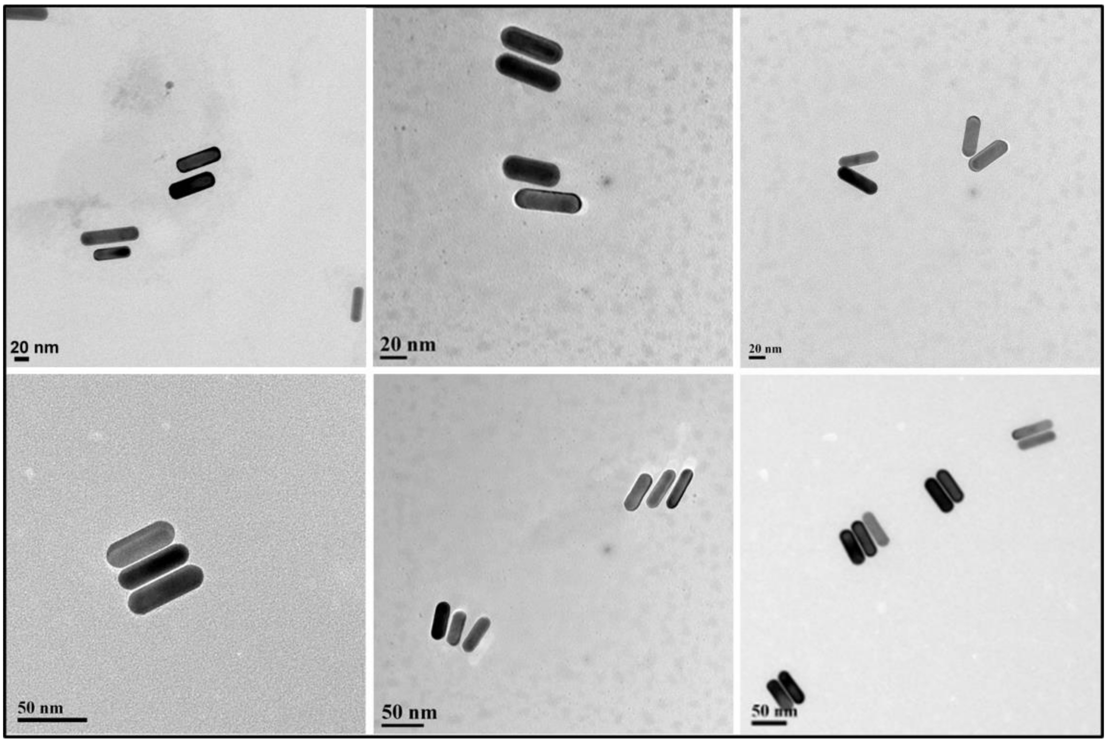

Supplementary Figure S7. Representative TEM images for SBS assembly after 2 PCR cycles.

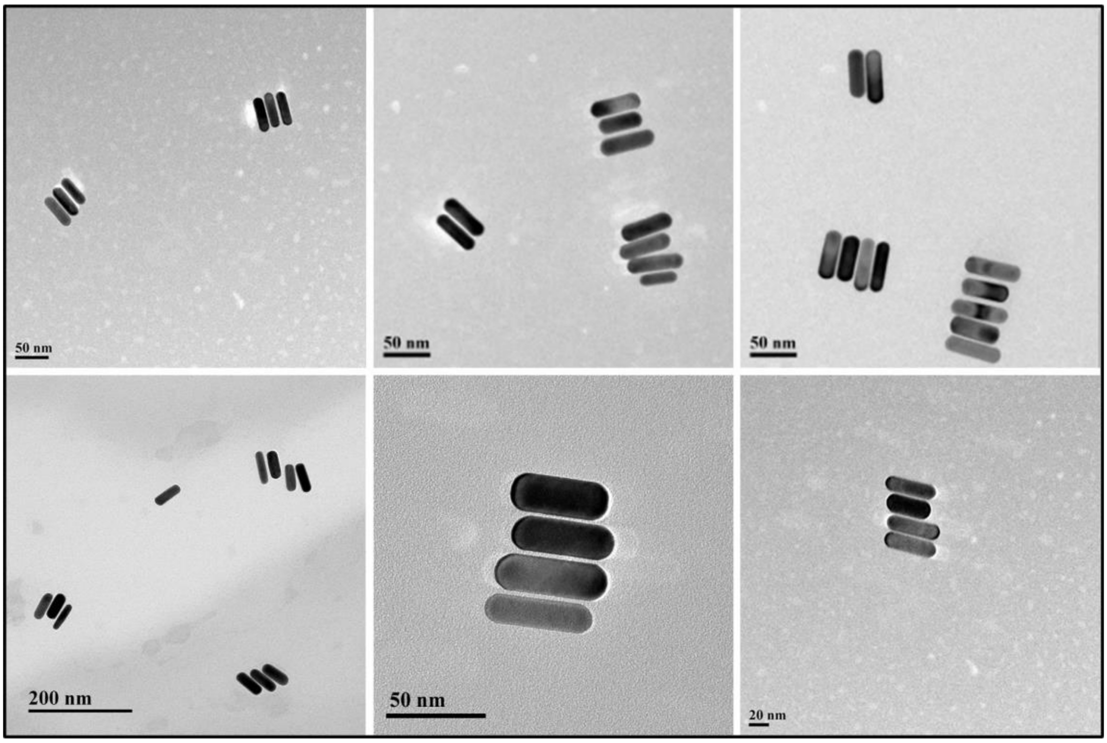

Supplementary Figure S8. Representative TEM images for SBS assembly after 5 PCR cycles.

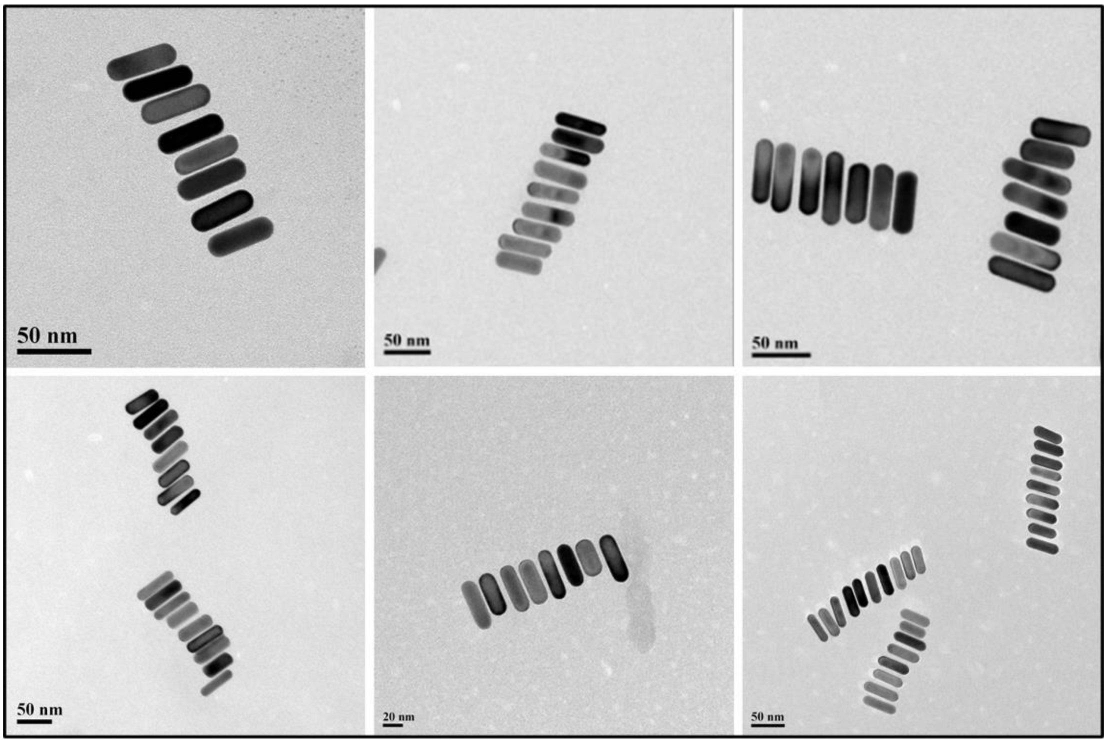

Supplementary Figure S9. Representative TEM images for SBS assembly after 10 PCR cycles.

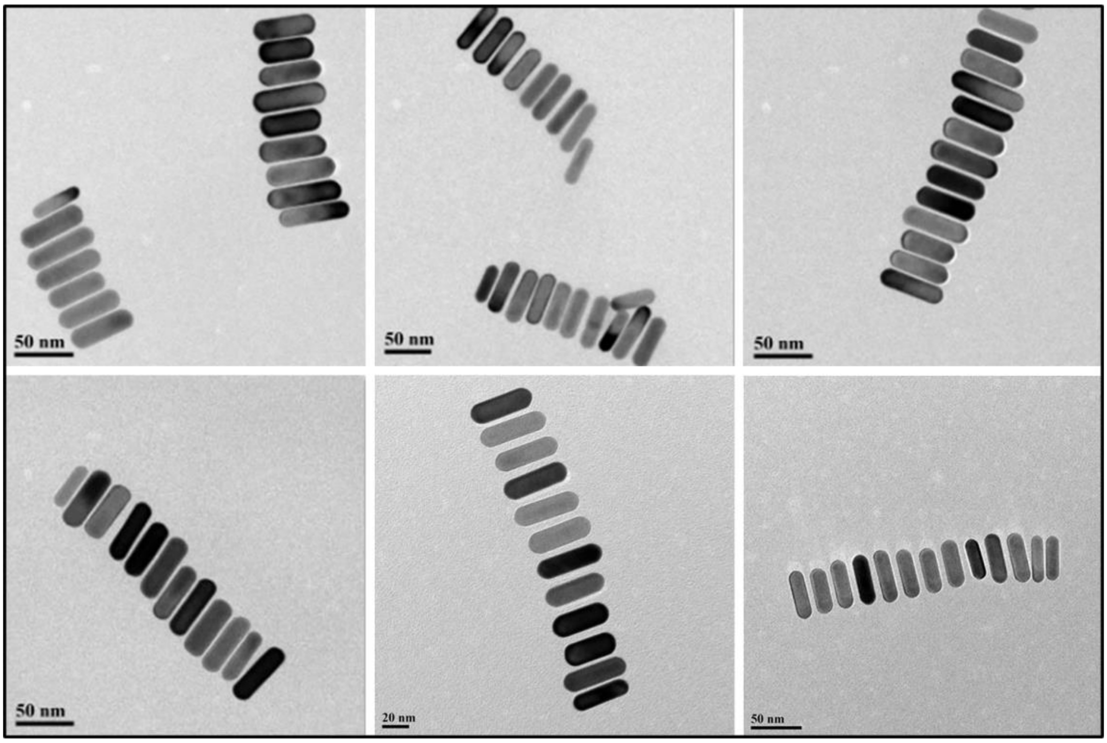

Supplementary Figure S10. Representative TEM images for SBS assembly after 15 PCR cycles.

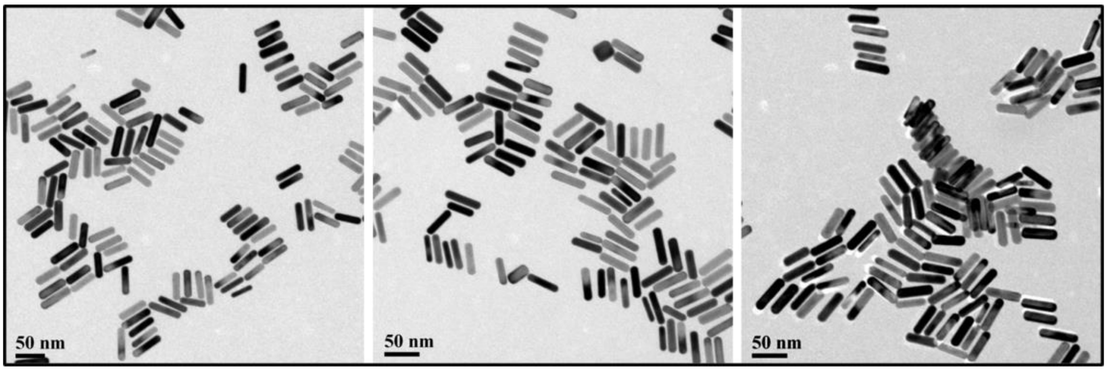

Supplementary Figure S11. Representative TEM images for SBS assembly after 20 PCR cycles.

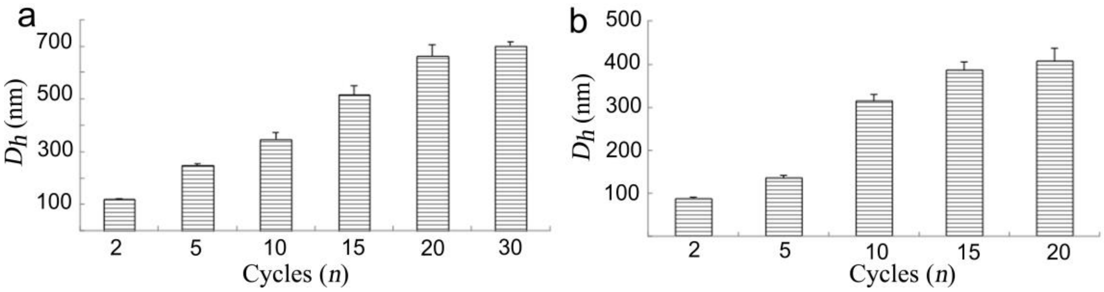

Supplementary Figure S12. The hydrodynamic diameters, D_h, of (a) ETE and (b) SBS assemblies obtained by dynamic light scattering (DLS) for different number of PCR cycles, n.

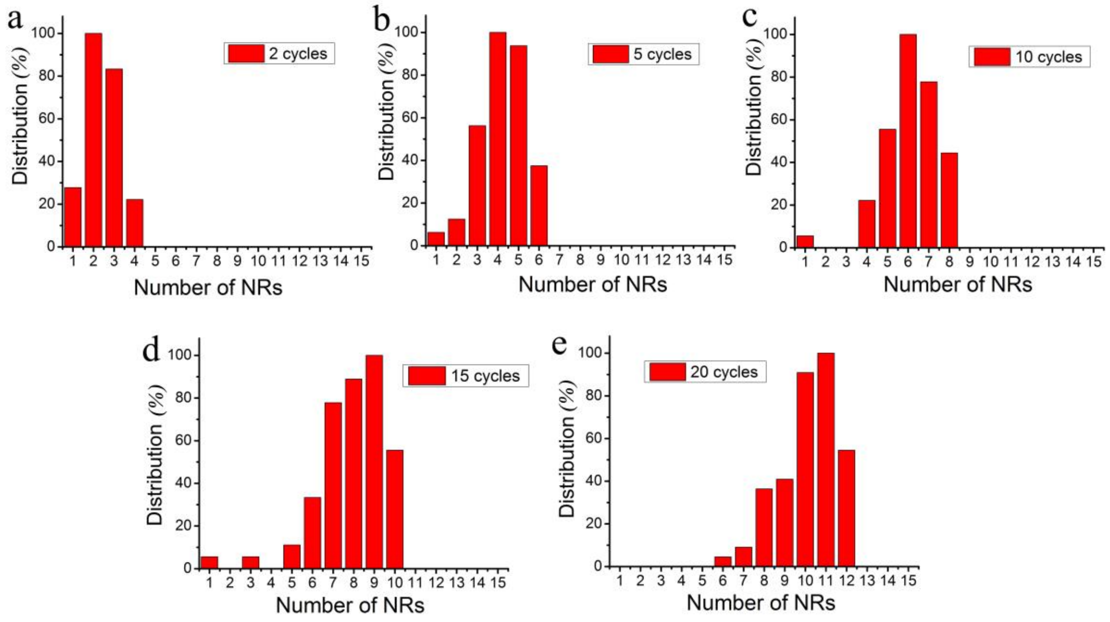

Supplementary Figure S13. Statistical analysis of number of NRs in ETE assemblies obtained after different numbers of PCR cycles using TEM images.

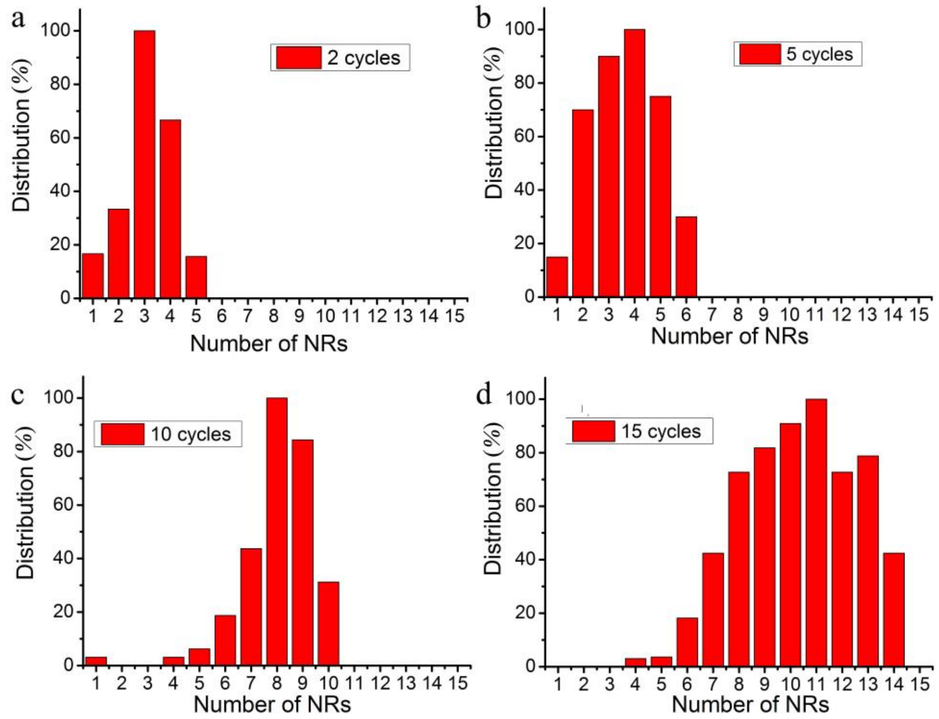

Supplementary Figure S14. Statistical analysis of number of NRs in SBS assemblies obtained after different numbers of PCR cycles using TEM images.

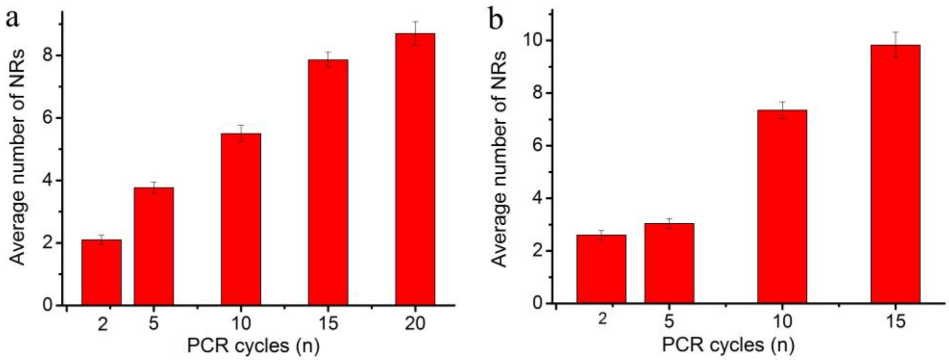

Supplementary Figure S15. The average number of NRs in (a) ETE and (b) SBS assemblies obtained after different numbers of PCR cycles, *n*.

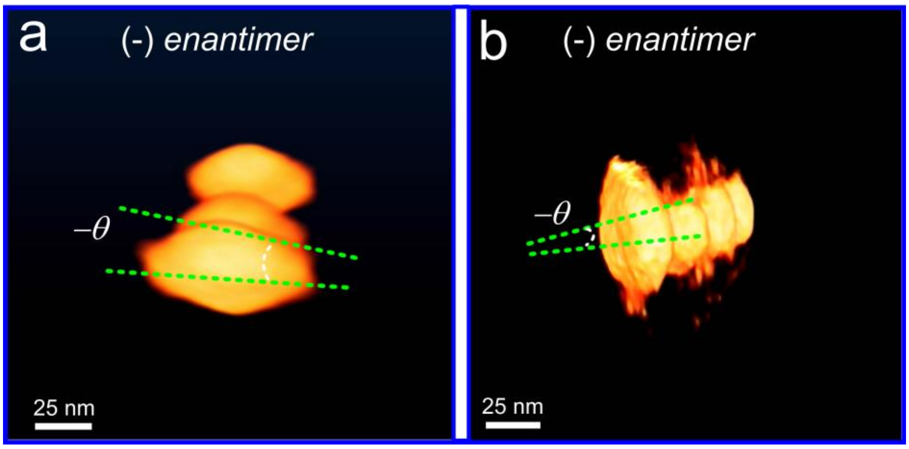

Supplementary Figure S16. Representative cryo TEM tomography image for (a) NR trimer and (b) tetramer. Twist angles were determined to be -7 deg and -8 deg for (a) and (b), respectively.

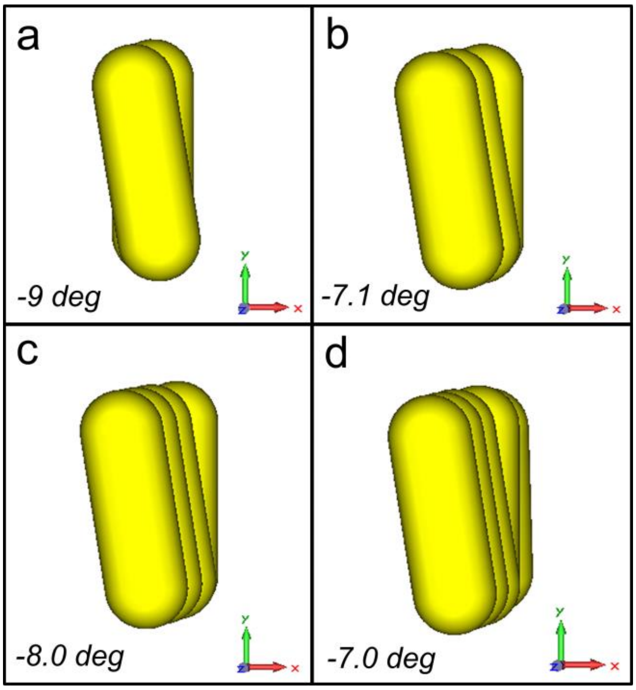

Supplementary Figure S17. Schematics of twisted dimer, trimer, tetramer, and pentamer according to TEM tomography with angles of -9.0, -7.1, -8.0, -7.0 deg used in the simulations of chirooptical properties of the DNA-bridged assemblies.

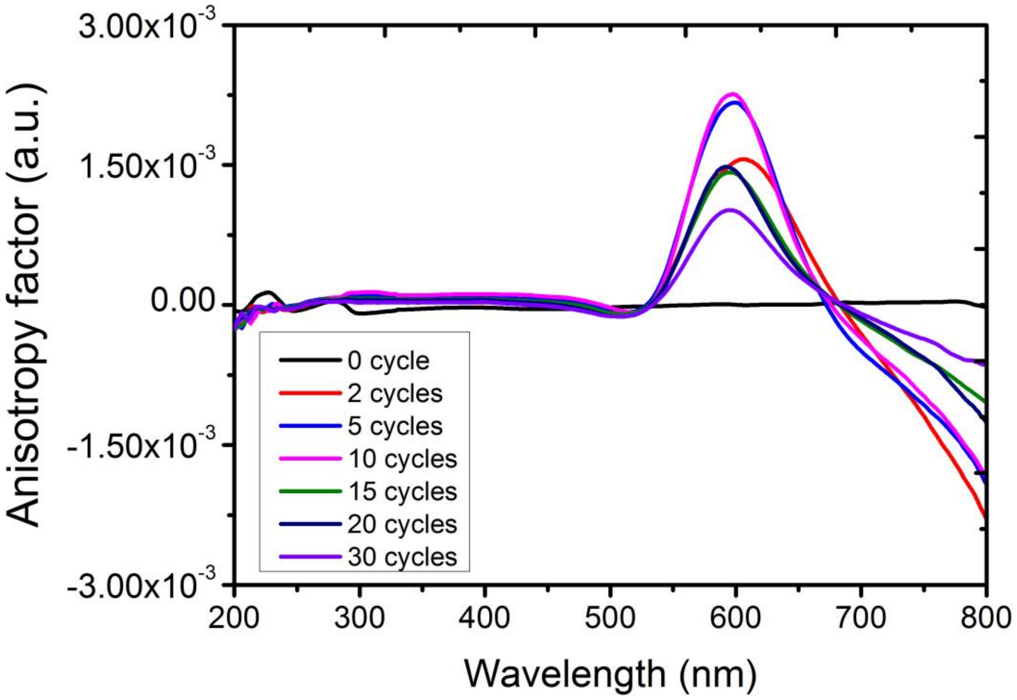

Supplementary Figure S18. Evolution of anisotropy factors (g) with the number of PCR cycles, n, from n = 0 to n = 30.

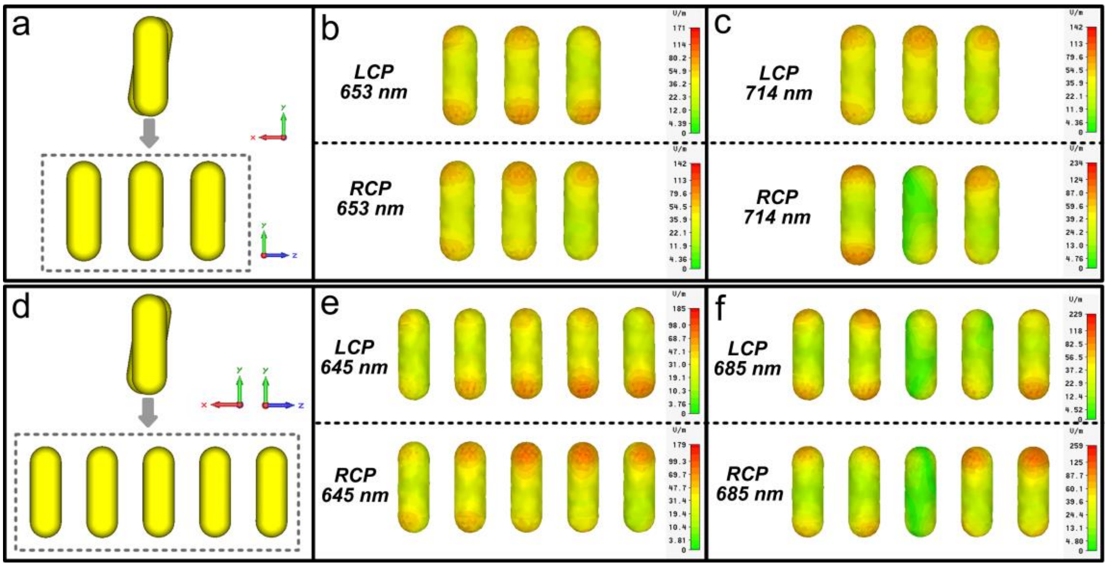

Supplementary Figure S19. Calculated maps of *E*-field on the surface of a chiral NR trimer and pentamer. (a) Schematics of a NR trimer used in the calculations. (b, c) Surface *E*-field distribution maps of NR trimer being excited at (b) 653 nm (positive band) and (c) 714 nm (negative band). (d) Schematics of a NR pentamer used in the calculations. (e, f) Surface *E*-field distribution maps of NR trimer being excited at (e) 645 nm (positive band) and (f) 685 nm (negative band).

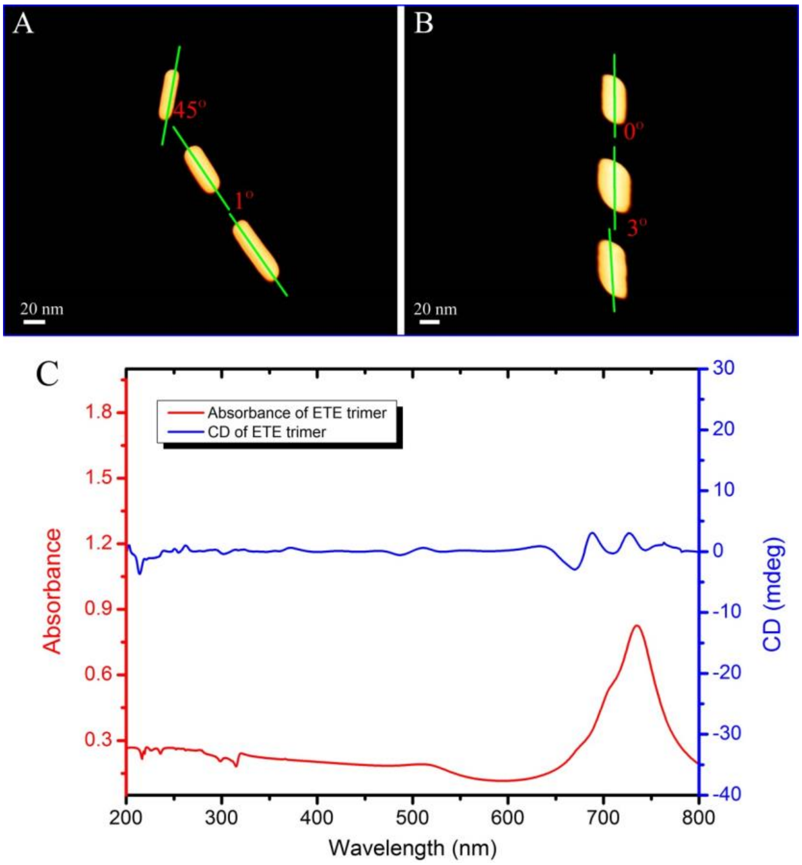

Supplementary Figure S20. The excitation beam was set under LCP and RCP excitation for a specific incident direction, z = 0 deg and φx = 90 deg. (a, b) Cryo TEM tomography images of ETE trimer and (c) the corresponding simulations of absorbance and CD spectra of the ETE assembly with 4π integration space.

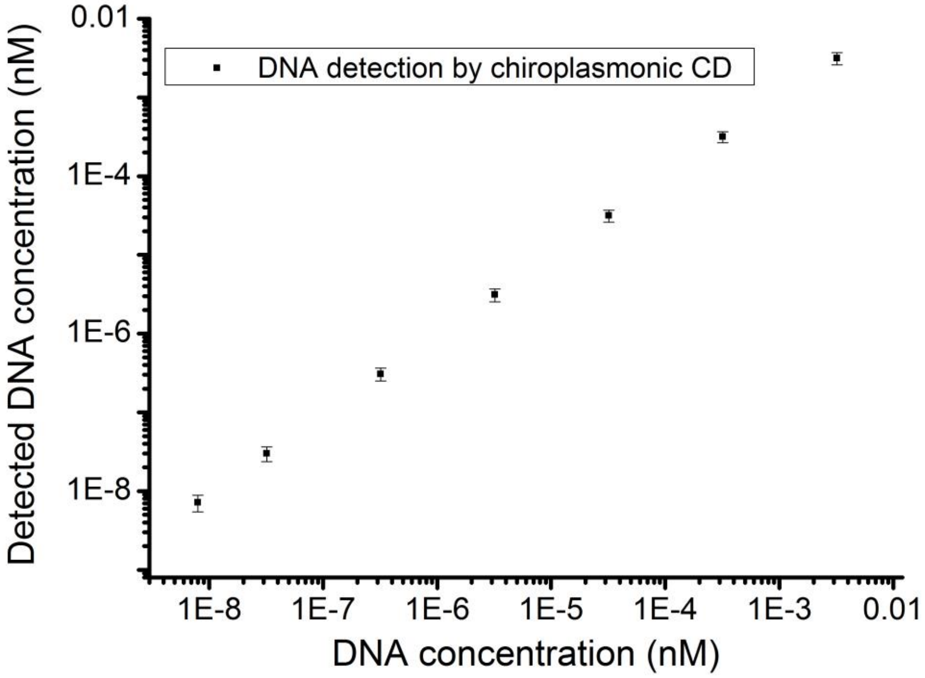

Supplementary Figure S21. Calibration of the DNA detection by the NR chiroplasmonic assembly method using the dilution method with *a priori* known concentrations of DNA.

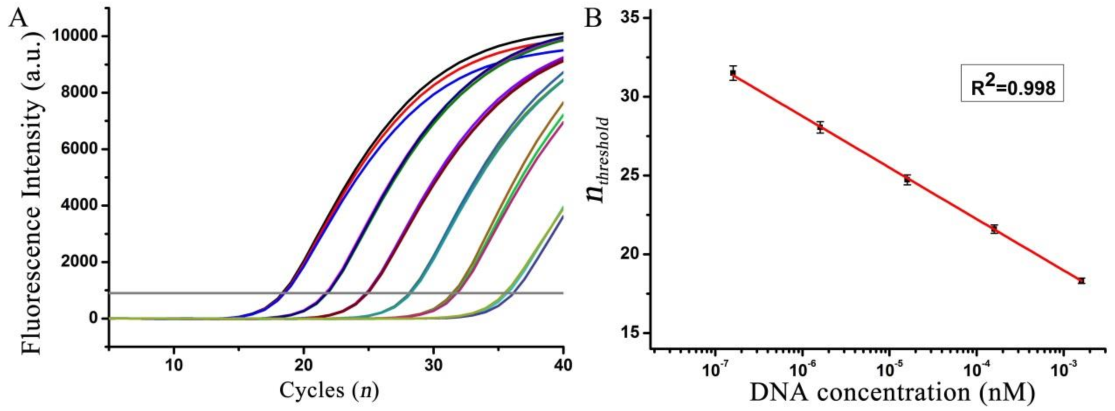

Supplementary Figure S22. (a) The original data obtained for DNA detection using the reverse transcription polymerase chain reaction (RT-PCR) ampilification method. The ampilification curves from left to right corespond to the 1.56 x10^{-3} nM with stepwise 10x dilution. (b) The standard RT-PCR calibration curve for concentrations of DNA with stepwise 10x dilution.

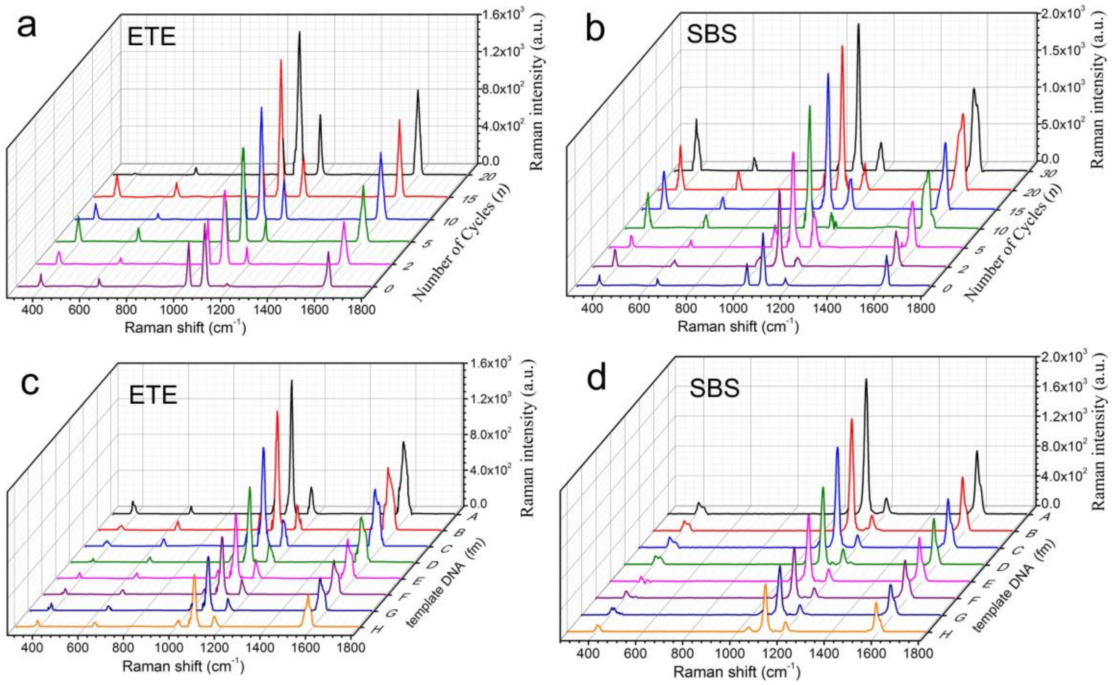

Supplementary Figure S23. (a, b) Experimental SERS spectra for DNA-bridged (a) ETE and (b) SBS assemblies with 4-ATM tag for different numbers of PCR cycles, *n*. (c, d) Experimental SERS spectra for DNA-bridged (c) ETE and (d) SBS assemblies with 4-ATP tag for series DNA concentrations with stepwise 10x dilution starting from 0.156 nM. SBS assemblies formed as the result of surface modification of NRs with polymeric molecules with hydrophobic tails can be successfully utilized for SERS sensing of Raman-active analytes.[31] Lee *et al*.[31] observed a continuous decrease of SERS and *E*-field intensity with increasing of number of NRs in SBS assemblies, while CD signal considerably increased with n for $0 < n < 10$.

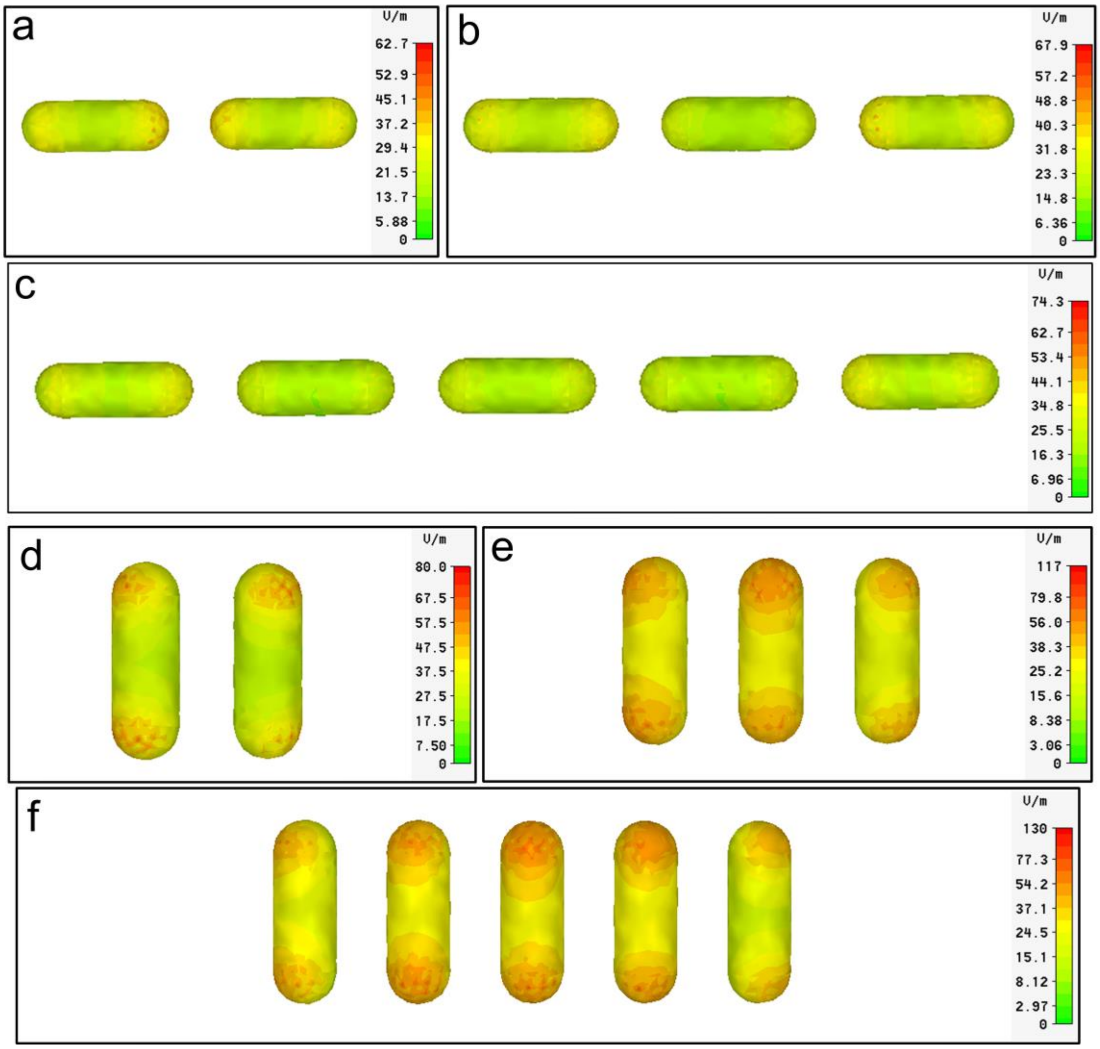

Supplementary Figure S24. (a, b, c) Calculated maps of *E*-field on the surface of NR (a) dimer, (b) trimer, and (c) pentamer assembled following the ETE pattern with linear configuration. The excitation wavelength is 632.8 nm. (d, e, f) Calculated maps of *E*-field on the surface of NR (d) dimer, (e) trimer, and (f) pentamer assembled following the SBS pattern. The excitation wavelength is 632.8 nm. The NR gap is 17.2 nm.

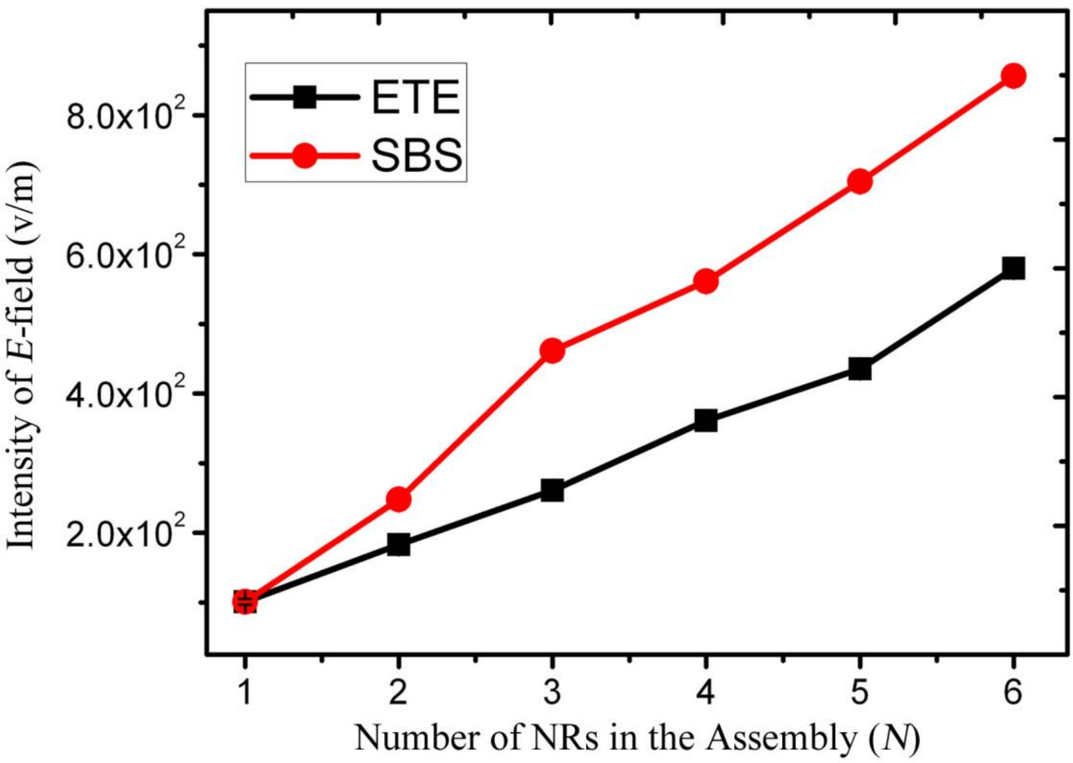

Supplementary Figure S25. The surface *E*-field for different number of Au NRs in DNA-bridged assemblies. The ETE was set as linear structure. The excitation wavelength was set as 632.8 nm, the gap was set as 17.2 nm, and twist angle as -9.0, -7.1, -8.0, -7.0 and -7.0 degrees, respectively. Differrent calculation runs used to determine *E*-field values give essentially identical values. The error bars are therefore not indicated in the plots.

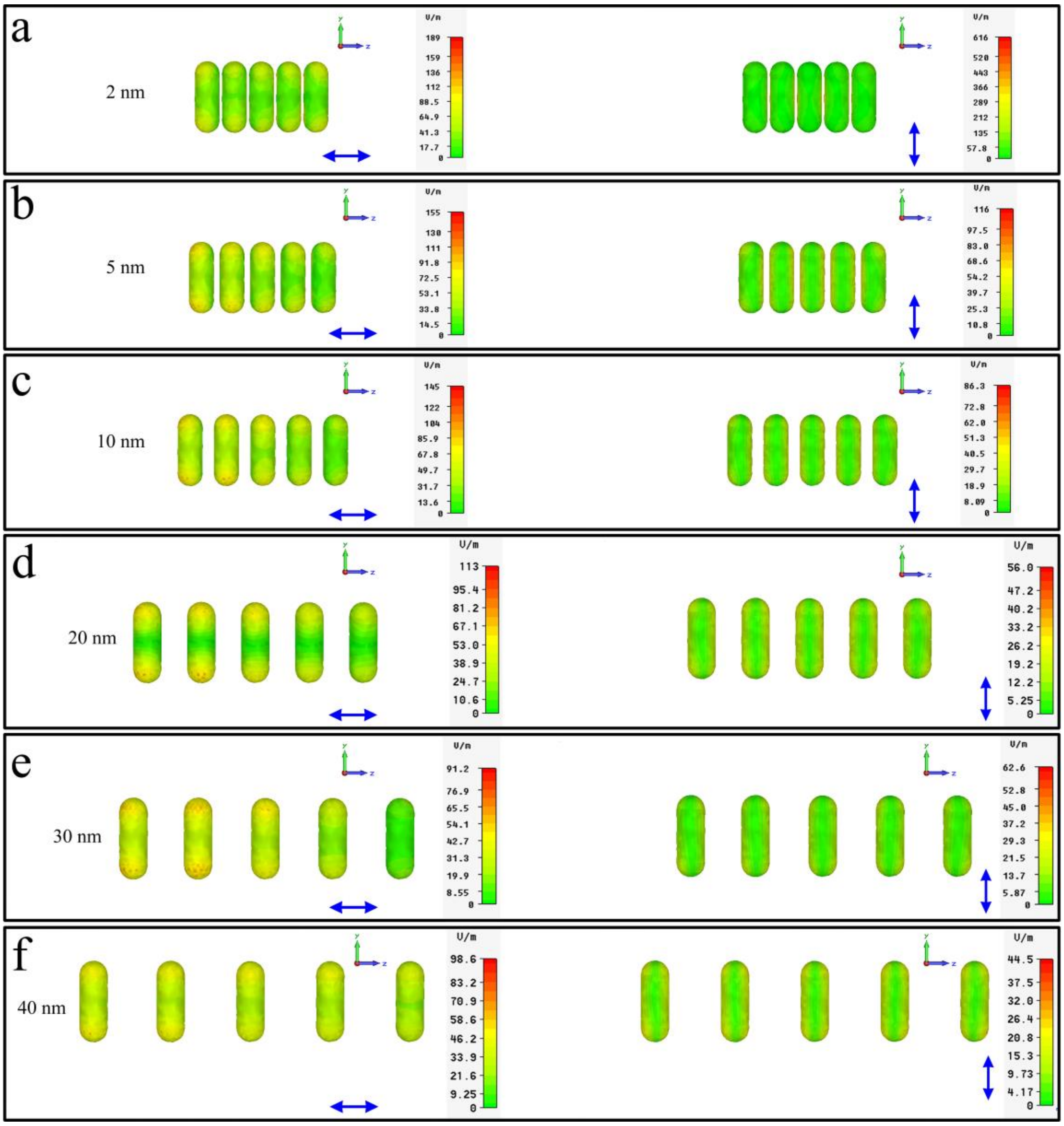

Supplementary Figure S26. Surface *E*-field distribution maps for NR pentamers with gaps of 2 nm, 5 nm, 10 nm, 20 nm, 30 nm, and 40 nm. The arrows demonstrate the *E*-field oscillation direction. Reported intensity represents the sum of the *E*-fields oscillating in both directions. The excitation wavelength is 632.8 nm. The twist angle is -7.0 deg.
